# Dual-channel NIR activatable theranostic prodrug for *in vivo* spatiotemporal tracking thiol-triggered chemotherapy†
†Electronic supplementary information (ESI) available: Detailed experimental procedures, characterizations, supplementary optical spectra and figures. See DOI: 10.1039/c6sc00970k


**DOI:** 10.1039/c6sc00970k

**Published:** 2016-04-28

**Authors:** Mingzhou Ye, Xiaohang Wang, Jianbin Tang, Zhiqian Guo, Youqing Shen, He Tian, Wei-Hong Zhu

**Affiliations:** a Key Laboratory of Biomass Chemical Engineering of Ministry of Education and Center for Bionanoengineering , College of Chemical and Biological Engineering , Zhejiang University , Hangzhou , Zhejiang 310027 , P. R. China . Email: jianbin@zju.edu.cn; b Key Laboratory for Advanced Materials and Institute of Fine Chemicals , Shanghai Key Laboratory of Functional Materials Chemistry , School of Chemistry and Molecular Engineering , East China University of Science and Technology , Shanghai 200237 , P. R. China . Email: whzhu@ecust.edu.cn ; Email: guozq@ecust.edu.cn

## Abstract

Real-time tracking for where (W), when (W), and how (H) prodrugs are delivered and activated *in vivo* is a great challenge for prodrug development. Disulfide linkage-based prodrugs as well as their delivery systems have been studied extensively, but the WWH question in spatial and temporal (spatiotemporal) precision remains unanswered. Herein, we present a novel prodrug of camptothecin (CPT) linked to a near-infrared (NIR) cyanine dye *via* a disulfide linkage (Cy-S-CPT). The cleavage of the disulfide bond in Cy-S-CPT by endogenous glutathione (GSH) can activate the anti-cancer drug CPT and induce a remarkable fluorescence shift from 825 to 650 nm, thereby providing dual fluorescent channels to real-time track the prodrug biodistribution and activation *in vivo*. Impressively, the dual-channel NIR fluorescence bioimaging exhibits the pervasive drug distribution, *i.e.*, the biodistribution of the intact prodrug was traced at the 825 nm-NIR fluorescence channel, whereas the activated drug was tracked at the 650 nm red fluorescence channel. In this way, we can overcome the blind spot in the metabolism kinetics of prodrugs in a certain organ or tissue. As demonstrated, the prodrug prompts activation in all the organs, particularly in the liver after an intravenous injection, and achieves predominant accumulation and activation in tumors at 24 h post injection. Cy-S-CPT loaded in PEG–PLA nanoparticles display significantly improved therapeutic efficacy and low side effects with respect to the clinical used drug CPT-11. As a consequence, the NIR spatiotemporal bioimaging *in vivo* with dual fluorescence channels allows the prodrug release profile to be extracted precisely, particularly in visualizing drug-released information from complex biological systems such as mice, thereby providing a unique opportunity to take insight into the relationship between theranosis and pharmacokinetics.

## Abstract 2

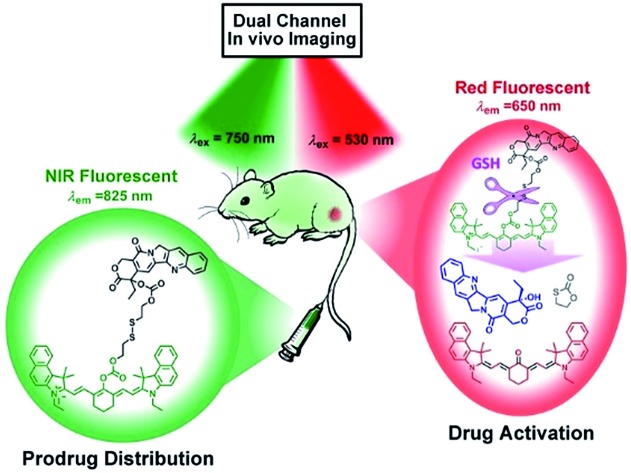
Real-time tracking of where, when, and how prodrugs are established. A novel theranostic prodrug based on the disulfide linkage with two distinct switchable near-infrared (NIR) fluorescence can precisely extract the prodrug release profile *in vivo* through dual-channel fluorescent imaging for the first time.

## Introduction

Traditional chemotherapy always suffers from undesired purgatorial side-effects, such as hair loss, bone marrow suppression, and organ damage, due to the inability to distinguish between cancer and normal cells.^1^ In contrast, tumor-specific prodrugs are activated precisely at the tumor sites by tumor-selective species,^2^ such as glutathione (GSH), enzymes or reactive oxygen, which are capable of greatly decreasing the off-target toxicity with an enhancement in the therapeutic index.^3^ Regarding the prodrugs, it is critical but difficult to track in real-time where (W), when (W), and how (H) they are delivered and activated *in vivo*.^4^ In particular, a large number of prodrugs and drug delivery systems based on the disulfide linkage have been developed, but their WWH questions in spatial and temporal (spatiotemporal) precision remain unanswered.^5^ Accordingly, theranostic prodrugs that integrate the modalities of therapy and diagnosis into a single “package” are of great interest to disclose the behavior of prodrugs in different individuals and provide an individualized or personalized treatment modality.^6^

Fluorescent biosensors can extract disease-related information precisely from complex biological systems due to their high sensitivity and resolution, low-cost, and real-time monitoring without radiopharmaceuticals.^7^ In particular, near-infrared (NIR) fluorescent bioimaging would be of particular interest for tracking the prodrug release profiles *in vivo*,^8^ because the long wavelength emission allows deep-tissue imaging, reduces auto-fluorescence and decreases light scattering.^9^ For instance, the dicyanomethylene-4*H*-pyran (DCM) chromophore and cyanine dyes have been well exploited as a NIR optical reporter for sensing the activation of theranostic prodrugs.^10^ However, all these established NIR fluorescent theranostic prodrugs suffer from only one turn-on a NIR readout channel after activation, *i.e.*, their NIR fluorescence was not observable before activation.^11^ In this way, because we are unable to simultaneously monitor the biodistribution and activation in spatiotemporal mode, the metabolism kinetics of prodrugs in a certain organ or tissue becomes a blind spot, which becomes a critical obstacle in precise diagnosis and chemotherapy.^12^ Undergoing spectrally distinct NIR fluorescence before and after prodrug-activation is expected to open a window to overcome the tough hurdle. However, using two-channel fluorescence for two-color NIR imaging *in vivo* to reveal the prodrug release profile has barely been investigated.

**Scheme 1:**
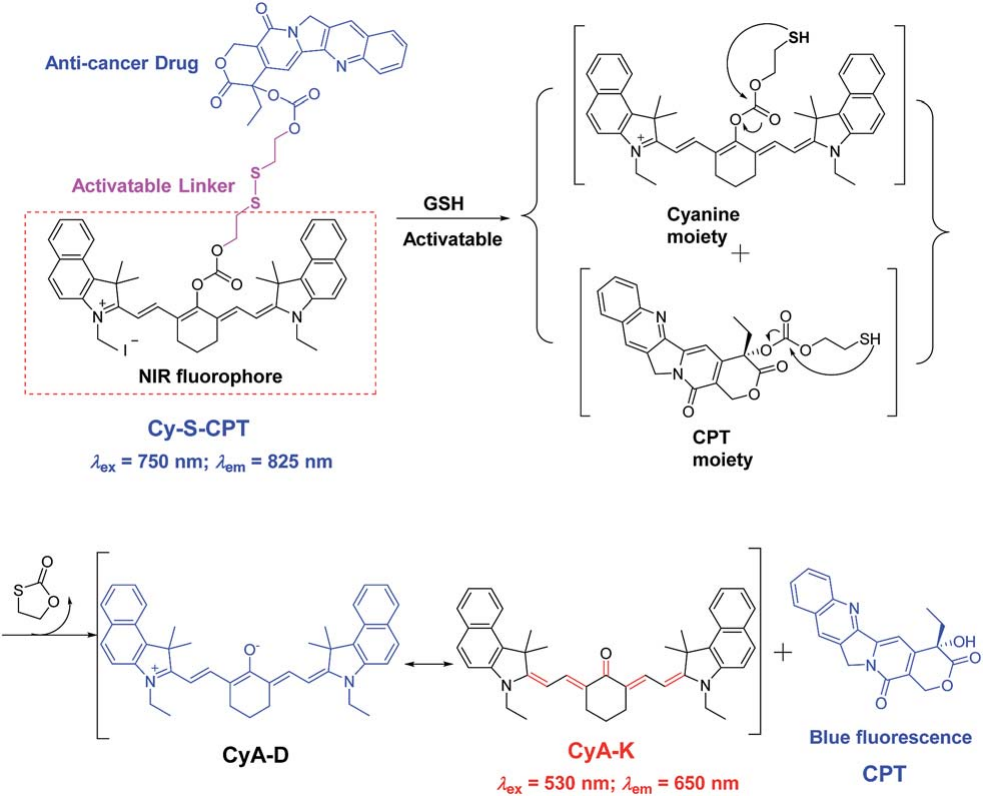
Proposed mechanism in CPT activation and fluorescent variation of the prodrug Cy-S-CPT by the treatment of GSH.

The key to developing dual-channel theranostic prodrug is to construct a bioactivatable NIR fluorescent reporter. Cyanine dyes have received immense attention as NIR fluorescent labels for biological applications.^13^ In particular, a remarkable spectral shift by modulating the pull–push conjugated π-electron system in cyanine chromophore makes them ideal fluorescent reporters for developing dual-channel prodrugs.^14^ Herein, we present a novel theranostic prodrug based on the disulfide linkage (Cy-S-CPT, Scheme 1) with two distinct switchable NIR fluorescence, enabling access to powerful theranosis *via* real-time tracking the prodrug activation and biodistribution *in vivo*. The specific cleavage of the disulfide bond by GSH and the successive cyclization produce the activated anti-cancer drug camptothecin (CPT) and induce a remarkable fluorescence shift from 825 to 650 nm, making a breakthrough with dual channels to spatiotemporally scrutinize the activation process of prodrugs *in vivo*.^15^ The NIR fluorescent bioimaging with two distinct channels *in vivo* was conducted on mice with a xenografted tumor to disclose the metabolism process of prodrugs with disulfide linkage, demonstrating a critical method of real-time tracking the delivery and activation process of prodrugs *in situ*. Moreover, the superior antitumor efficacy of the prodrug delivered by a polymeric nanoparticle was revealed, along with achieving high efficacy and low side-effects.

## Results and discussion

### Synthesis of NIR fluorescent prodrug

As depicted in Scheme S1 in the (ESI†), Cy-S-CPT was obtained by a three-step procedure. First, CyA-E was prepared by the reaction of cyanine dye IR-739 with sodium acetate in *N*,*N*-dimethylformamide (DMF) with a yield of about 64%. The key intermediate CPT-S-OH was synthesized in two steps: first the anticancer drug of CPT reacted with triphosgene in chloroform, then a solution of 2,2′-disulfanediylbis(ethanol) in CHCl_3_–THF mixture was added dropwise to generate CPT-S-OH. The final product, Cy-S-CPT, was obtained from the treatment between CPT-S-OH and CyA-E in the presence of triphosgene at room temperature. Moreover, as a control, a non-cleavable linker (“C–C”) was used instead of a disulfide linker (“S–S”) to synthesize Cy-C-CPT under the similar synthetic strategy (Scheme S1†). To improve their solubility and pharmacokinetics, Cy-S-CPT or Cy-C-CPT was both loaded in polyethylene glycol–polylactic acid (PEG–PLA) nanoparticles, which is a drug carrier with excellent biocompatibility, and has been approved for clinical use in Korea.^16^ The prodrug-loaded nanoparticle, PEG–PLA/Cy-S-CPT, had an average size of around 80 nm measured by dynamic laser scattering (DLS, Fig. S1†) and revealed a slight negative charge with a zeta potential of –4.6 mV. The drug loading of the PEG–PLA nanoparticle for Cy-S-CPT was 5.8% w/w, and the encapsulation efficiency was 67.3% w/w. The nanoparticles PEG–PLA/Cy-C-CPT had an almost identical size and zeta potential as those of PEG–PLA/Cy-S-CPT. All the detailed procedures and characterization are shown in ESI.†

### Dual fluorescence with GSH-activatable response

**Fig. 1:**
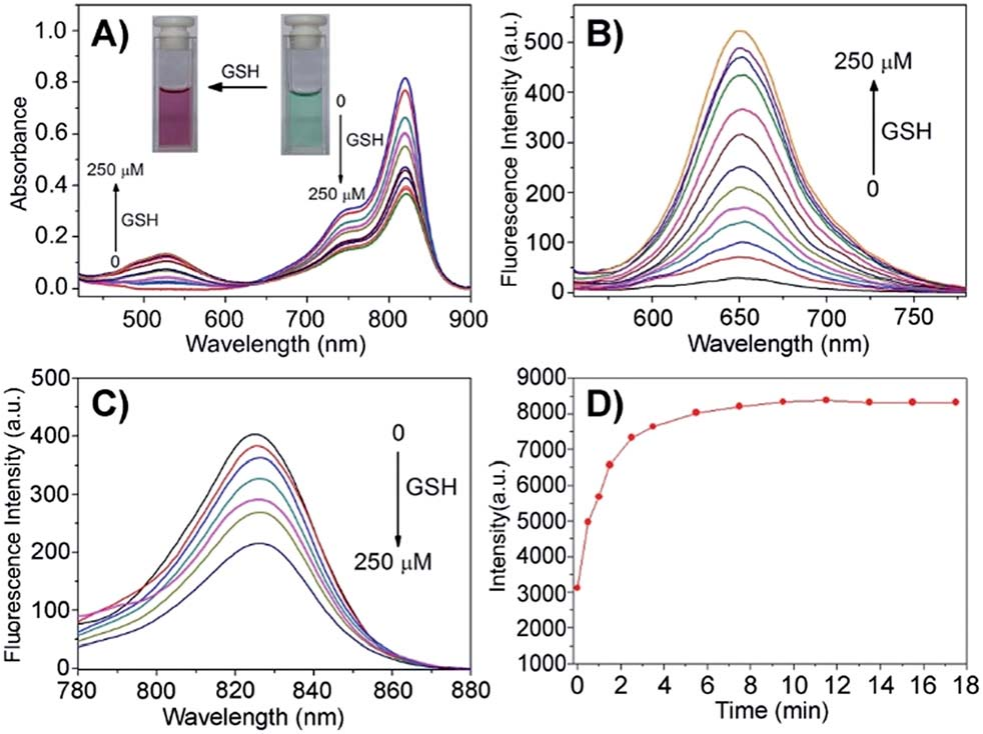
GSH-activatable response of prodrug Cy-S-CPT (5 μM) with the titration of GSH (0, 1, 5, 10, 15, 25, 35, 50, 75, 100, 150 and 250 μM) and incubation for 15 min in a mixture solution of DMSO/PBS (40/60, pH = 7.4, 10 mM) at 37 °C: spectral change in absorption (A) and emission ((B), *λ*_ex_ = 530 nm; (C), *λ*_ex_ = 750 nm), and time dependence of fluorescence intensity (D) at 650 nm in the presence of GSH (250 μM) upon excitation at 530 nm.

Given that the disulfide bond is a well-known activatable linker by thiols,^17^ the following experiments of prodrug Cy-S-CPT were first carried out to test our cleavage hypothesis of a disulfide linker by GSH. To examine the response of Cy-S-CPT to GSH, the prodrug Cy-S-CPT (5 μM) was titrated with various concentrations of GSH in the mixed DMSO/PBS solution (40/60, v/v, pH = 7.4, 10 mM). As expected, the prodrug Cy-S-CPT produced both colorimetric and fluorescence spectral changes upon the addition of GSH. Upon the addition of 250 μM of GSH, Cy-S-CPT exhibited a remarkable shift change in the absorption spectra, along with color change from green to purple red (Fig. 1A). The absorption peak at 810 nm (*ε* = 1.9 × 10^5^ M^–1^ cm^–1^) decreased sharply, and a new band centered at 530 nm (*ε* = 2.8 × 10^4^ M^–1^ cm^–1^) was observed, along with a distinct isosbestic point at about 635 nm. Concomitantly, a large hypsochromic shift in the emission spectra was also observed, *i.e.*, a NIR band at 825 nm in the emission spectra decreased with the excitation at 750 nm (Fig. 1B), and a sharp increase at 650 nm was found with excitation at 530 nm (Fig. 1C), within 10 min to reach reaction equilibrium at 37 °C (Fig. 1D). It is likely that the distinct emission spectral change of Cy-S-CPT in the presence of GSH is a consequence of the disruption in the polymethine π-electron system, *i.e.*, the pull–push π-conjugation system in the tricarbocyanine chromophore is obviously shortened, resulting in a large hypsochromic shift in both the absorption and emission spectra.^18^ More importantly, the absorbance ratio (*A*_535 nm_/*A*_820 nm_) in this activatable prodrug exhibited a linear response with the GSH concentration (0–100 μM, Fig. S2†).

Subsequently, the spectra of CyA-K were compared with Cy-S-CPT treated with GSH (Fig. 1 and S3†). The identical positions and shapes in both absorption and emission indicated that CyA-K was the resulting product upon the addition of GSH to the Cy-S-CPT system. In contrast, the control Cy-C-CPT with alkane bonds (“C–C”) as a linker displayed nearly no change in the photophysical properties upon treatment with GSH (Fig. S4†). Clearly, these distinct spectroscopic response differences between Cy-S-CPT and Cy-C-CPT upon GSH indicated that the optical response of Cy-S-CPT as well as the release of CPT was derived from the disulfide cleavage by GSH (Scheme 1). Moreover, the expected released CPT as an active cancer drug and the corresponding intermediates were further confirmed by ESI-MS analyses. Upon the addition of GSH with Cy-S-CPT, the ionic peaks of 593.3493 (corresponding to [CyA-K + H]^+^) and 453.1734 (corresponding to [CPT moiety + H]^+^) in Scheme 1 were observed simultaneously in the high resolution MS spectrum (HRMS, Fig. S5†), clearly indicating that the active CPT could be released from the prodrug Cy-S-CPT, with the concomitant generation of the NIR reporter CyA-K by a two-step process (a cleavage of disulfide bond and then intramolecular cyclization).^19^

The performance of Cy-S-CPT upon other biologically relevant analytes, such as amino acids and abundant metal ions, was also investigated, as shown in Fig. S6.† Owing to their thiol-containing structures, similar spectroscopic responses of Cy-S-CPT to that with GSH were observed with 1,4-dithiothreitol (DTT), cysteine (Cys) and homocysteine (Hcy). On the other hand, no appreciable fluorescence change with the spectral shift was induced by the treatment of other non-thiol amino acids and metal ions, confirming the specific cleavage of a disulfide bond elicited by thiol-containing species. Actually, in real biological systems, there is a 100–1000 fold difference in the GSH concentration between cell cytoplasm and body fluids (2–10 mM *versus* about 2 μM), and tumor cells possess even higher GSH levels than normal cells.^20^,^21^ In contrast, the aforementioned potential interferences of DTT, Cys and Hcy could be neglected because they always have a relatively low concentration, which is in contrast to the high concentration of GSH in the cytoplasm.^22^ Accordingly, we can explore both the emission bands of prodrug Cy-S-CPT (825 nm) and the resulting CyA-K (650 nm) as a dual-channel NIR prodrug upon the specific GSH-induced disulfide cleavage in drug-delivery systems, together with high selectivity over other various potential biological interference, including metal ions.

### Cellular uptake and distribution

**Fig. 2:**
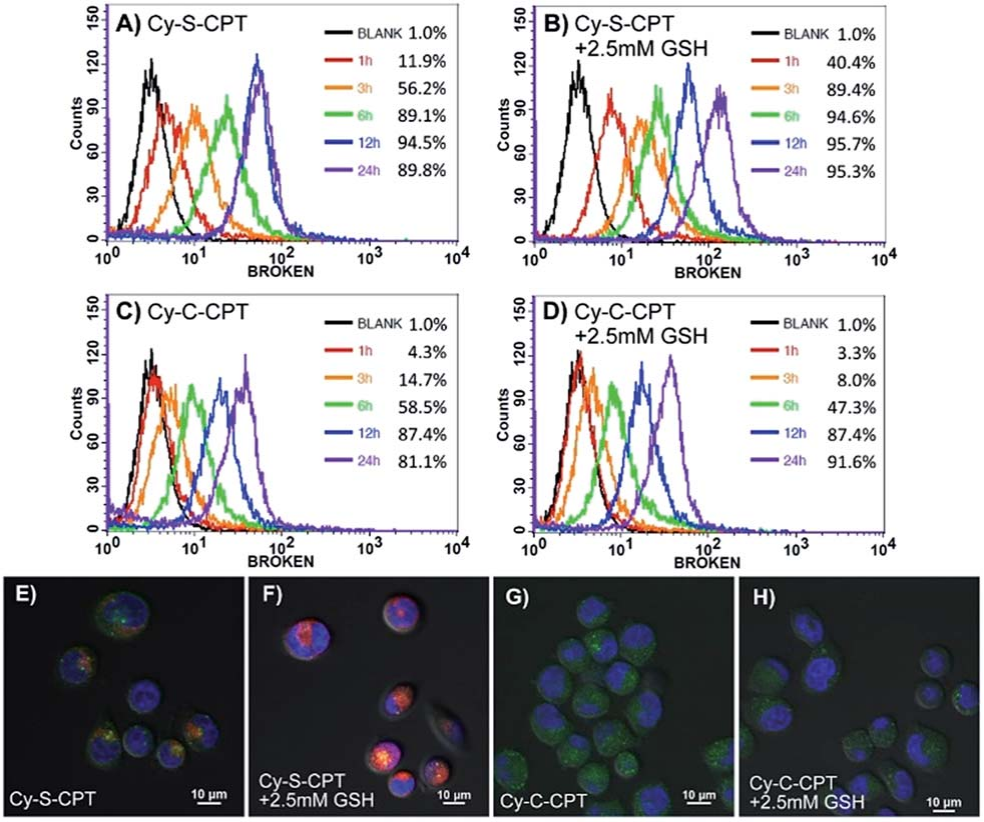
Cellular uptake and intracellular trafficking of prodrugs Cy-S-CPT and Cy-C-CPT in BCap-37 cells: flow cytometry analysis of the cellular uptake and activation for Cy-S-CPT (A and B) and Cy-C-CPT (C and D) at different time intervals from 1 to 24 h with and without extra 2.5 mM GSH, and confocal laser scanning microscopy images of BCap-37 cells cultured with Cy-S-CPT or Cy-C-CPT with (E and G) or without (F and H) extra GSH for 2 h. The *X*-axis is FL-3 channel that captured the fluorescence of CyA-K (*λ*_ex_ = 530 nm, *λ*_em_ = 650 nm); the nucleus was stained with DAPI and lysosome was stained by Lysotracker green. Note: red signal indicates the fluorescence from CyA-K (*λ*_ex_ = 530 nm, *λ*_em_ = 650 nm) representing the prodrug activation by GSH.

The cellular uptake and activation of the prodrug were further monitored by flow cytometry. The cells with the activated prodrug and concurrent CyA-K with deep-red fluorescence at 650 nm were counted at different times. As shown in Fig. 2A–D, the ratios of the cells with fluorescence increased from 11.9% to 89.1% upon increasing the incubation time from 1 to 6 h, which is indicative of a gradual cellular uptake and activation of prodrug. This procedure could be largely accelerated by additional 2.5 mM GSH, reaching 40.4% at 1 h and 89.4% at 3 h, respectively. In contrast, the group incubated with Cy-C-CPT showed a much lower ratio of cells with red fluorescence, and GSH barely promoted activation of Cy-C-CPT (Fig. 2C and D), also demonstrating that the interaction between the disulfide bond and GSH was the major driving force of CPT release in the cells.^23^

To confirm the finding by flow cytometry, as well as intuitively exhibit the cellular uptake and activation of prodrug, confocal laser scanning microscopy was further employed to observe BCap-37 cells treated with Cy-C-CPT and Cy-S-CPT. As shown in Fig. 2E–H, there was abundant CyA-K fluorescence (red) distributed in the cytoplasm after 2 h incubation with Cy-S-CPT, which is indicative of the quick uptake and intracellular activation of the prodrug. In addition, the florescence in cells was enhanced significantly by an extra 2.5 mM GSH, which further proved the cleavage by GSH. On the other hand, the cells treated by Cy-C-CPT either with or without GSH showed very rare CyA-K florescence, suggesting that most prodrugs were still in the intact status, and the disulfide linkage was indeed critical for prodrug activation.^24^

### Cytotoxicity evaluation

**Fig. 3:**
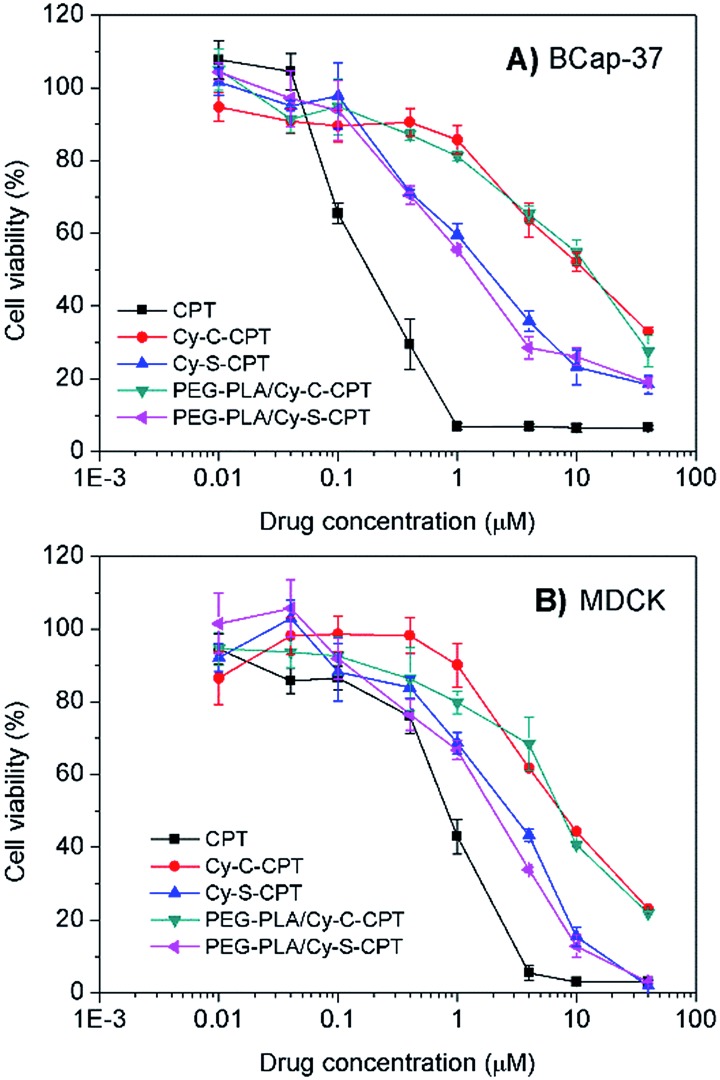
Cytotoxicity of Cy-S-CPT, Cy-C-CPT, PEG–PLA/Cy-S-CPT, PEG–PLA/Cy-C-CPT and CPT against BCap-37 cancer cells (A) and MDCK normal cells (B).

The cytotoxicity against cancer cell BCap-37 and normal MDCK lines was investigated using a standard MTT assay (Fig. 3). As expected, the prodrug Cy-S-CPT with the intracellular activation revealed much higher antitumor activity *in vitro* than Cy-C-CPT. The IC_50_ value (the concentration inhibiting cell growth to 50% of control) of Cy-S-CPT was 1.7 μM, which is in contrast to 11.3 μM of Cy-C-CPT, but slightly higher than their parent drug CPT. Intriguingly, the prodrug loaded in the PEG–PLA nanoparticles (PEG–PLA/Cy-S-CPT) had identical cytotoxicity against cancer cells to the free prodrugs, which was favorable for prodrug delivery by polymeric nanoparticles. However, similar cytotoxicities were also observed on MDCK cells for the prodrugs, suggesting that the prodrug could be activated in normal cells such as MDCK cells, and thus selective accumulation in tumor with a delivery system was required to decrease the side effects on the normal tissue.^25^

### Pharmacokinetic study

**Fig. 4:**
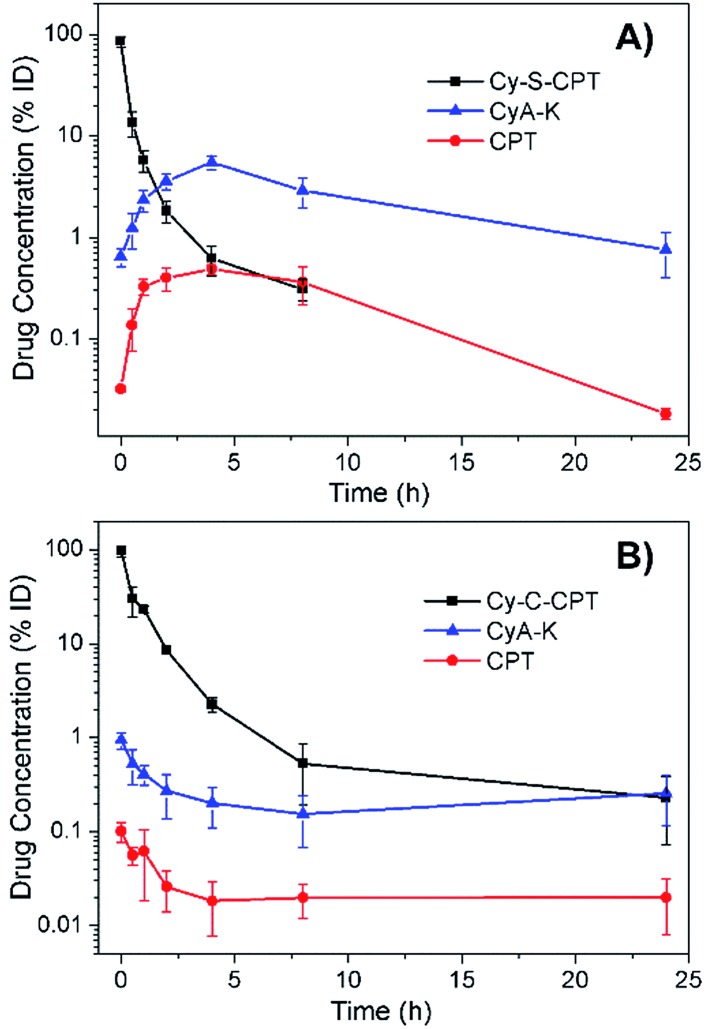
Blood concentrations of Cy-S-CPT, CyA-K and CPT as a function of time after a single intravenous injection of PEG–PLA/Cy-S-CPT (A) and PEG–PLA/Cy-C-CPT (B) at a dose of 10 μmol kg^–1^. Note that the absent data point at 24 h means its blood concentration was lower than the detection limit.

For the prodrug loaded in the PEG–PLA nanoparticles, the blood clearance of PEG–PLA/Cy-S-CPT and PEG–PLA/Cy-C-CPT was measured after a single intravenous administration at a dose equivalent to 10 μmol kg^–1^ CPT (Fig. 4). In contrast to the fast clearance of CPT,^26^ the clearance of PEG–PLA/Cy-S-CPT became much slower. More intriguingly, while the concentration of Cy-S-CPT in the blood decreased with the lasting time, the concurrently formed CyA-K was enhanced with the prodrug activation in the blood and reached its peak of a 5.5% injected dose at 4 h post-injection. This was 8.8 times higher than the concentration of the intact prodrug. However, the concurred concentration of CyA-K decayed with the further aging time due to the decreasing activation of Cy-S-CPT and fast clearance of CyA-K. Clearly, the activation of Cy-S-CPT *in vivo* was fast with the maximal activation around 4 h after the injection. In contrast, for the group injected with PEG–PLA/Cy-C-CPT, the concentration of Cy-C-CPT in the blood decreased with the time due to the clearance, but there was no increase in the concentration of CyA-K and CPT observed.

### *In vivo* imaging and biodistribution in spatial and temporal precision

**Fig. 5:**
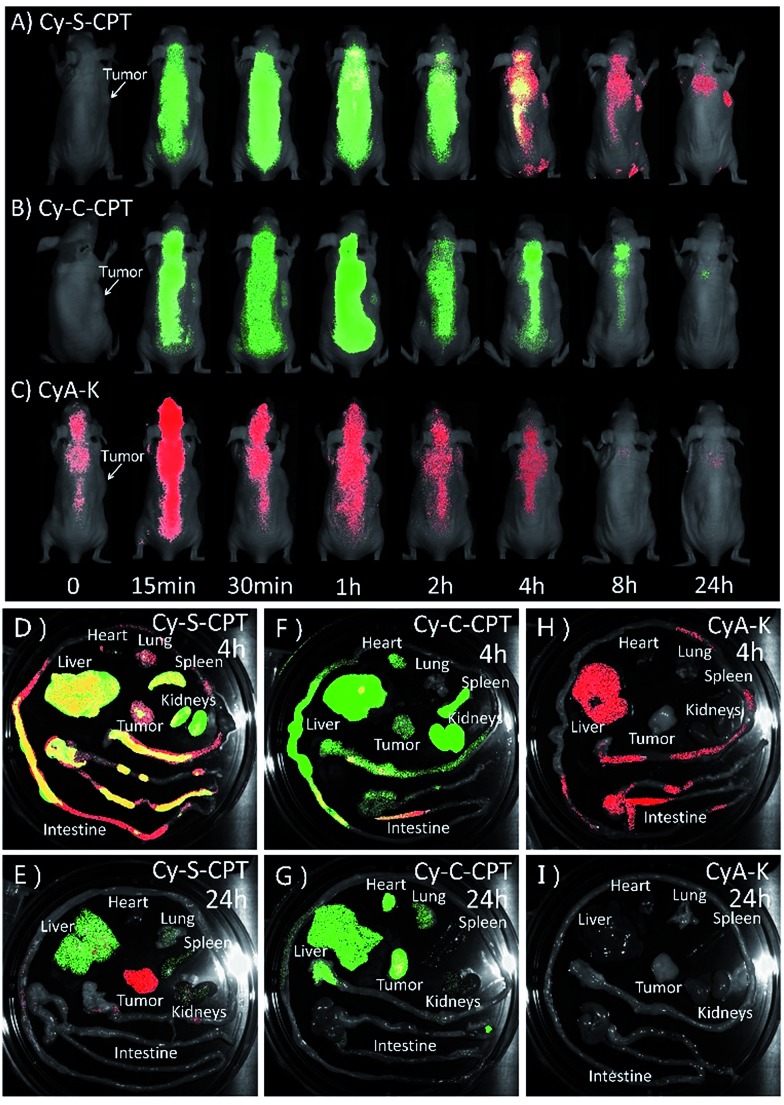
Biodistribution of PEG–PLA loaded prodrugs. *In vivo* and *ex vivo* bioimaging of tumor-bearing mice at various time (0.25, 0.5, 1, 2, 4, 8 and 24 h) after intravenous injection of (A) PEG–PLA/Cy-S-CPT, (B) PEG–PLA/Cy-C-CPT and (C) PEG–PLA/CyA-K at a dose of 5 μmol kg^–1^. Fluorescent images of the internal anatomy organs for the mice injected with PEG–PLA/Cy-S-CPT (A and B), PEG–PLA/Cy-C-CPT (C and D) and PEG–PLA/CyA-K (E and F) at 4 or 24 h after an intravenous injection. Note that the green signal represents the fluorescence from the intact prodrugs (*λ*_ex_ = 750 nm, *λ*_em_ = 825 nm) for tracking the prodrug biodistribution, whereas red signal represents the fluorescence from CyA-K (*λ*_ex_ = 530 nm, *λ*_em_ = 650 nm) for tracking the location of drug activation. Yellow represents the coexistence of prodrug and drug release.

To date, all reported NIR fluorescent theranostic prodrugs suffered from only one turn-on readout channel. In regard to *in vivo* bioimaging, the biodistribution of prodrugs before activation in a certain organ or tissue is difficult to be recognized or monitored. In the prodrug Cy-S-CPT, the remarkable blue shift in fluorescence from CyA-E (*λ*_em_ = 825 nm) to CyA-K (*λ*_em_ = 650 nm, Scheme 1) made it possible to simultaneously track or visualize the biodistribution and activation of the prodrugs through the specific dual fluorescent channels. The *in vivo* and *ex vivo* fluorescent bioimaging of mice after the intravenous injection of the prodrug loaded PEG–PLA nanoparticles was performed with different channels. In this way, the biodistribution of the intact prodrug was traced at the 825 nm-NIR fluorescence channel (represented in green), whereas the activated drug was tracked at the 650 nm red fluorescence channel (represented in red). The overlap of green and red shown in yellow indicates that both the prodrug and activated prodrug co-existed at the same place. As shown in Fig. 5A, the NIR fluorescence was quickly distributed in the whole body of mice, indicating a fast distribution of Cy-S-CPT loaded nanoparticles, and the NIR fluorescence gradually faded out due to the activation and excretion of the prodrug. Simultaneously, the red fluorescence became stronger with time before 4 h post injection due to the gradual prodrug activation, and it faded out as time was further increased owing to the excretion of the activated drug. Intriguingly, even though the activation occurred in all organs, the tumor bestowed the strongest red fluorescence at 650 nm channel, which is relative to the activation of the prodrug, particularly 24 h post injection.

Consistent with the finding of the *in vivo* study, the *ex vivo* study also showed that Cy-S-CPT accumulated and released CPT in all the main organs, particularly in the liver, at 4 h, and abundant fluorescence in both colors was allocated in the intestine canal, validating that bowel evacuation is an important path for the prodrug clearance (Fig. 5D). Notably, almost all the prodrug accumulated in the intestinal contents rather than in the intestinal wall, indicating the prodrug cannot be absorbed by the digestive system and there was no enterohepatic circulation in the prodrug metabolic process (Fig. S7†). At 24 h after intravenous injection, most of the prodrug and drug were eliminated with some remnant in the liver and tumor in different forms. While there existed abundant NIR fluorescence signal in the liver, the tumor region exhibited strong red fluorescent signals, indicative of a continuous CPT release that triggered by plentiful GSH in the tumor (Fig. 5E). For comparison, the biodistribution of the Cy-C-CPT-loaded nanoparticles was also checked. It can be seen that the NIR fluorescence was distributed in the whole mice, but deep-red fluorescence was hardly observed, once again manifesting that Cy-C-CPT without a disulfide bond could barely be activated in mice (Fig. 5B, F and G). In addition, there was no selective accumulation in the tumor for the mice injected with the CyA-K loaded nanoparticles both from the *in vivo* and *ex vivo* bioimaging (Fig. 5C, H and I), through which we can rule out the possibility that the activated drug and CyA-K were transported into the tumor after their activation and the resulting false positive in the image.

To obtain a deeper insight into the drug distribution in tumor, the confocal images of tumor slices sectioned from mice treated with PEG–PLA/Cy-S-CPT are displayed in Fig. S8.† Red signals that represented CyA-K deep-red fluorescence increased in the tumor tissues from 2 to 24 h, which illustrated the gradual drug activation process in the tumor. Notably, the relatively uniformed fluorescence spread in the entire view at 24 h, indicating that the prodrug could penetrate deep into the tumor tissues and release drugs that affect the whole area and thus possess preferable antitumor effectiveness.

Both the *in vivo* and *ex vivo* imaging as well as the pharmacokinetic study indicated that the biodistribution and activation of Cy-S-CPT were visualized successfully through the dual-channel fluorescent imaging in the spatial and temporal mode. More importantly, it is clarified that the prodrug with disulfide could be activated not only in the tumors, but also in other organs, particularly the liver.^27^ The finding also semi-quantitatively gave the metabolism kinetics of the prodrug with a disulfide bond, which was found with the highest prodrug activation level at *ca.* 4 h after intravenous injection. Moreover, the tumor exhibited the highest prodrug accumulation and the most everlasting activation, which was probably due to its higher GSH concentration.

### *In vivo* antitumor activity

**Fig. 6:**
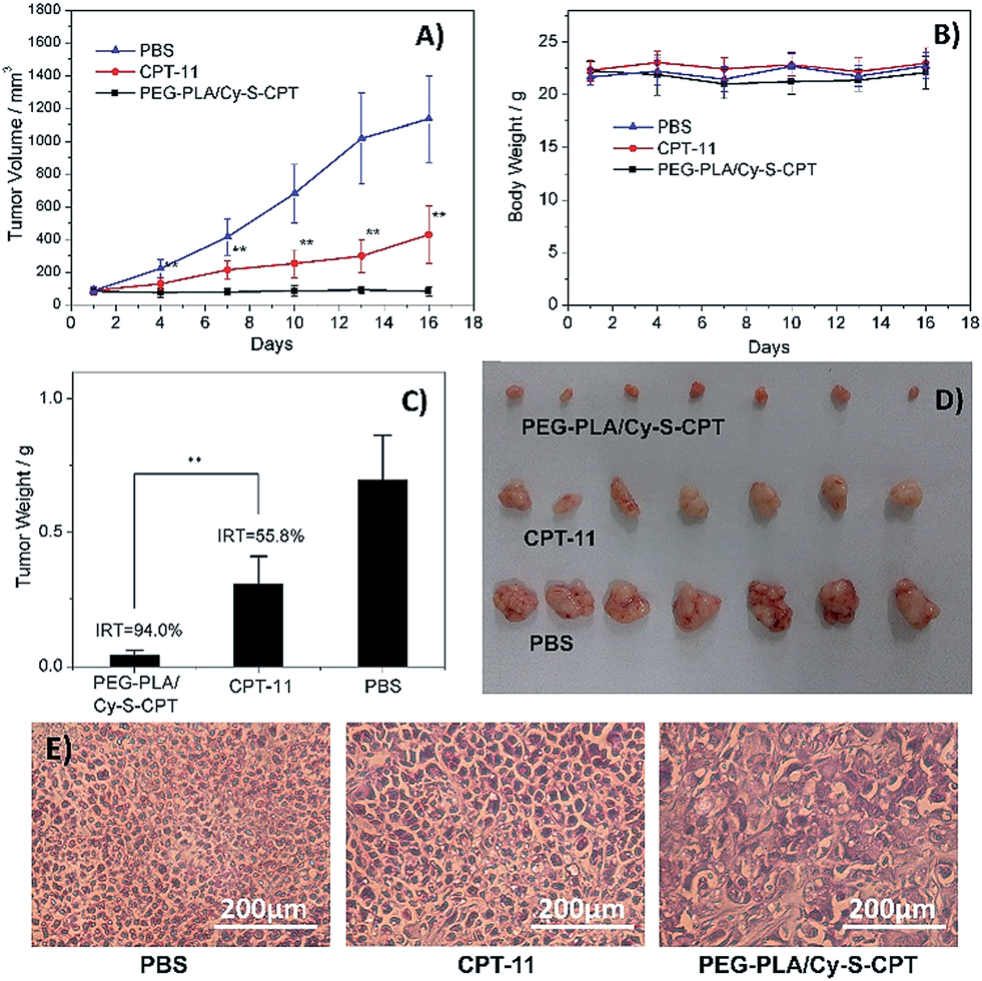
Antitumor activities of PBS, CPT-11 and PEG–PLA/Cy-S-CPT intravenously administered at a CPT-equivalent dose of 10 mg kg^–1^ every 3 days, as indicated by the arrows, against BCap-37 xenograft tumors (*n* = 7, data expressed as average ± SE, * or ** means *P* < 0.05 or 0.01, respectively). The results are summarized as the tumor volumes of mice bearing BCap-37 tumors exposed to various treatments (A), the body weight changes (B), the tumor weights of each group of the mice at the end of the experiment and inhibition rates of tumor growth (IRT, C), the image of tumors (D), and representative histological features of BCap-37 tumors from the treated mice (E).

The *in vivo* therapeutic efficacy of the prodrug Cy-S-CPT was evaluated in comparison with the clinically used irinotecan (CPT-11) and PBS on BCap-37 tumor xenograft model. Mice were injected intravenously with PEG–PLA/Cy-S-CPT, CPT-11 and PBS at a CPT equivalent dose of 10 mg kg^–1^ with an intermittent q2d × 5 schedule, respectively. Both CPT-11 and PEG–PLA/Cy-S-CPT could inhibit BCap-37 tumor growth compared to the blank control group, showing low toxicity with no significant weight loss during treatment (Fig. 6A and B). It can be noted that PEG–PLA/Cy-S-CPT exhibited much higher antitumor efficacy than CPT-11. A significant statistical difference in tumor volume was observed between the two groups merely 3 days after treatment (*P* < 0.01) and lasted until the end of the therapy. The average tumor volume in the CPT-11 group was multiplied 4.8 times at 16 days after the first injection, whereas PEG–PLA/Cy-S-CPT group, by comparison, did not show any increase in tumor volume. At the end of these experiments, all the tumors were collected and weighed to calculate the inhibition rate of tumor growth (IRT). PEG–PLA/Cy-S-CPT revealed an IRT as high as 94.0%, substantially exceeding 55.8% of CPT-11 (Fig. 6C), which was intuitively demonstrated by the tumor images shown in Fig. 6D. The higher antitumor efficacy of PEG–PLA/Cy-S-CPT was probably due to the higher tumor accumulation from the enhanced permeability and retention effect of the nanoparticle, as well as the efficient activation of Cy-S-CPT in the tumor (Fig. 5).

A further histological study was conducted on the tumor tissue sections with H&E staining. Compared to the tightly and regularly packed spherical cells in the PBS control group, the cells in the groups treated by CPT-11 and PEG–PLA/Cy-S-CPT swelled and exhibited severe vacuolization, which were typical apoptotic characteristics. Moreover, the tumors treated with PEG–PLA/Cy-S-CPT also had significantly lower tumor cellularity and more apoptotic cells than those treated with CPT-11 (Fig. 6E), which was consistent with the higher IRT induced by PEG–PLA/Cy-S-CPT than CPT-11.^28^

## Conclusions

We developed a novel theranostic prodrug Cy-S-CPT with switchable NIR fluorescence from 825 to 650 nm, providing dual fluorescent channels to real-time *in vivo* visualize the activation and biodistribution process of the prodrug with a disulfide linkage. In regard with the two distinct NIR fluorescence channels, we can not only real-timely tracked where the intact prodrugs are located *in vivo*, but also visualized when and how the active prodrugs were released and delivered to the cancer sites. The flow cytometry assay and confocal microscopy observation showed that in contrast to Cy-C-CPT, which mainly kept intact in the cancer cells, Cy-S-CPT was quickly activated by the intracellular GSH. The dual-channel *in vivo* pharmacokinetic and biodistribution study indicated that the prodrug with disulfide could be activated not only in the tumors, but also in the other organs, particularly the liver. Moreover, Cy-S-CPT loaded in the PEG–PLA nanoparticles (PEG–PLA/Cy-S-CPT) revealed an inhibition rate of tumor growth as high as 94.0%, substantially exceeding 55.8% of the clinical used drug CPT-11. As demonstrated, PEG–PLA/Cy-S-CPT bestowed significantly improved therapeutic efficacy and low side effects. We believe that the prodrug with different NIR fluorescence upon activation provides a new platform to explore the mysterious behaviors of the prodrugs with dual-channel fluorescent imaging *in vivo*.

## Supplementary Material

Supplementary information
