# Supplementary material for: Dual-channel NIR activatable theranostic prodrug for in vivo spatiotemporal tracking thiol-triggered chemotherapy
Source: Chem Sci. 2016 Apr 28;7(8):4958–65. doi: 10.1039/c6sc00970k (PMC6018301; doi:10.1039/c6sc00970k)
Supplement: Supplementary file 1 [file SC-007-C6SC00970K-s001.pdf]

Electronic Supplementary Material (ESI) for Chemical Science.
This journal is © The Royal Society of Chemistry 2016

Electronic Supplementary Information

Dual-channel NIR activatable theranostic prodrug for *in vivo* spatiotemporal tracking thiol-triggered chemotherapy†

Mingzhou Ye,[‡a] Xiaohang Wang,[‡b] Jianbin Tang,*[a] Zhiqian Guo,*[b] Youqing Shen,[a] He Tian[b] and Wei-Hong Zhu*[b]

[a]Key Laboratory of Biomass Chemical Engineering of Ministry of Education and Center for Bionanoengineering, College of Chemical and Biological Engineering, Zhejiang University, Hangzhou, Zhejiang 310027, P. R. China. E-mail: jianbin@zju.edu.cn.

[b]Key Laboratory for Advanced Materials and Institute of Fine Chemicals, Shanghai Key Laboratory of Functional Materials Chemistry, School of Chemistry and Molecular Engineering, East China University of Science and Technology, Shanghai 200237, P. R. China. E-mail: whzhu@ecust.edu.cn; guozq@ecust.edu.cn.

[‡]These authors contributed equally to this work.

Contents

1. Experimental section......S2
2. Synthetic route to Cy-S-CPT and Cy-C-CPT S2
3. Size distribution of PEG-PLA/Cy-S-CPT and PEG-PLA/Cy-C-CPT......S10
4. Absorbance ratio of Cy-S-CPT as a function of GSH concentration. S11
5. Normalized absorption and emission spectra of CyA-K and Cy-S-CPT S11
6. Fluorescence spectra of Cy-S-CPT with and without GSH S12
7. HRMS spectrum of Cy-S-CPT with 50 equiv of GSH S13
8. Fluorescence response of Cy-S-CPT with various thiol-containing structures and amino acids S13
9. Intestinal wall and intestinal contents images of mice injected prodrugs......S14
10. Prodrug penetration kinetics in tumor......S15
11. ^{1}H and ^{13}C NMR, and HRMS S15

1. Experimental section

All solvents were of analytical grade. ^1H and ^{13}C NMR in $CDCl_3$ were obtained by a Bruker AV-400 spectrometer with tetramethylsilane (TMS) as internal standard. High Resolution Mass Spectra (HRMS) were obtained by a Waters LCT Premier XE spectrometer. Absorption spectra were measured on a Varian Cary 500 spectrophotometer at 37 °C. Fluorescence spectra were recorded on a Varian Cary Eclipse fluorescence spectrophotometer (1 cm quartz cell) at 37 °C. Deionized water was used to prepare all aqueous solutions.

2. Synthetic route to Cy-S-CPT and Cy-C-CPT

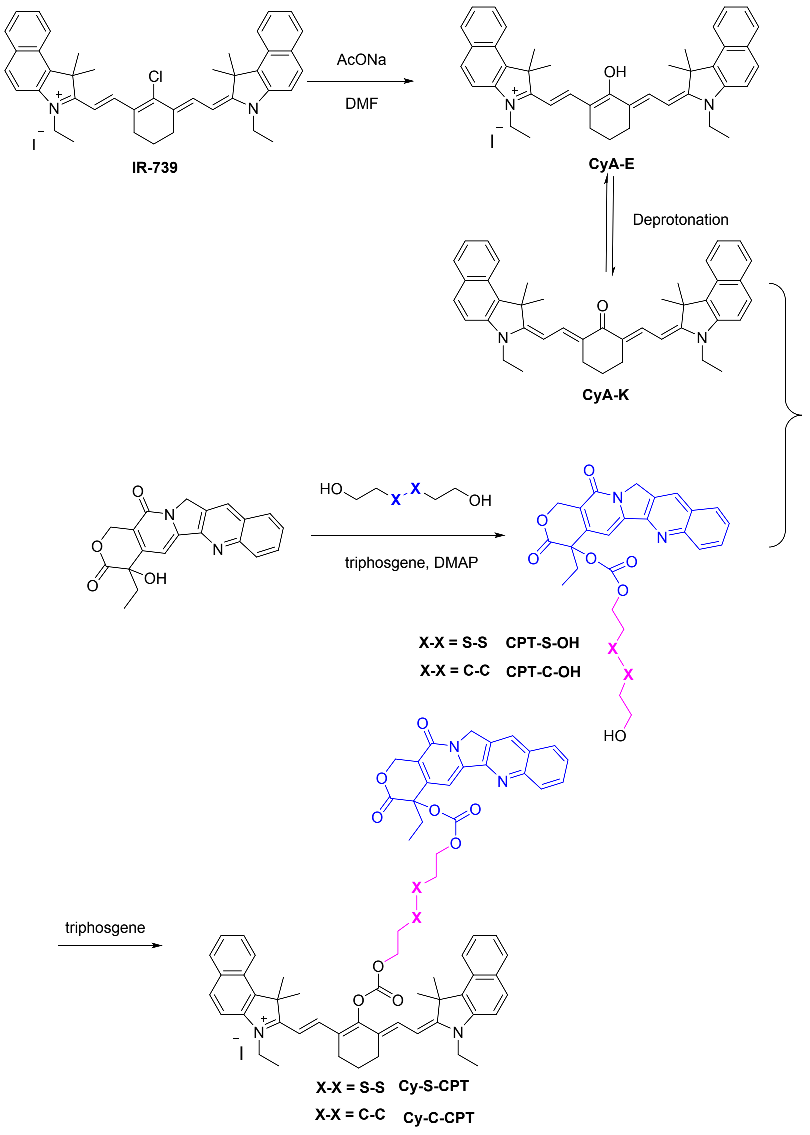

Scheme S1. Synthetic route to Cy-S-CPT and Cy-C-CPT

Synthesis of CyA-K

A mixture of IR-739 (311 mg, 0.41 mmol) and sodium acetate (125 mg, 1.49 mmol) in 15 mL *N*,*N*-dimethylformamide was heated at 90 °C for 6 h under Ar atmosphere. The solvent was removed by rotary evaporation to obtain red oil product, and then the product was purified by column chromatography (silica gel column, dichloromethane: triethylamine = 100 : 1) to obtain a red powder (210 mg), yield 64 %. ^{1}H NMR (400 MHz, $CDCl_3$, ppm): δ 1.33 (t, J = 7.2 Hz, 6H, -$NCH_2C\boldsymbol{H}_3$), 1.93-1.87 (m, 2H, -$C\boldsymbol{H}_2$-), 2.01 (s, 12H, -$C\boldsymbol{H}_3$), 2.66 (t, J = 5.6 Hz, 4H, -$C\boldsymbol{H}_2$-$C\boldsymbol{H}_2$-), 3.85 (q, J = 7.2 Hz, 4H, -$NC\boldsymbol{H}_2CH_3$), 5.51 (d, J = 13.4 Hz, 2H, alkene-H), 7.07 (d, J = 8.8 Hz, 2H, Ph-H), 7.27 (t, J = 7.2 Hz, 2H, Ph-H), 7.47 (t, J = 8.4 Hz, 2H, Ph-H), 7.76 (d, J = 8.8 Hz, 2H, Ph-H), 7.80 (d, J = 8.2 Hz, 2H, Ph-H), 8.04 (d, J = 8.4 Hz, 2H, Ph-H), 8.32 (d, J = 13.4 Hz, 2H, alkene-H). ^{13}C NMR (100 MHz, $CDCl_3$, ppm): δ 11.50, 22.63, 25.90, 27.93, 29.24, 31.45, 36.50, 37.17, 48.59, 91.79, 109.00, 121.86, 122.50, 126.33, 126.80, 129.41, 129.92, 132.76, 141.07, 162.55, 163.6, 186.28. HRMS (ESI): calcd. for $[C_{42}H_{44}ON_2 + H]^+$ 593.3532; found 593.3528. (Figure S6-8).

Synthesis of CPT-S-OH

A mixture of CPT (100 mg, 0.29 mmol), DMAP (122 mg, 1 mmol) and triphosgene (64 mg, 0.42 mmol) 10 mL of anhydrous chloroform was stirred for 1 h at room temperature, then a solution of 2,2'-disulfanediylbis(ethan-1-ol) in $CHCl_3$:THF (1 : 1, 5 mL) was added dropwise. The mixture was stirred overnight at room temperature. After the removal of the solvent by rotary evaporation, the product was purified by column chromatography (silica gel column, dichloromethane : methanol = 50 : 1) to get a white powder (120 mg), yield 78 %. ^{1}H NMR (400 MHz, $CDCl_3$, ppm): δ 1.02 (t, J = 7.2 Hz, 3H, -$C\boldsymbol{H}_3$), 2.24-2.12 (m, 1H, -$C\boldsymbol{H}_2CH_3$), 2.36-2.24 (m, 1H, -$C\boldsymbol{H}_2CH_3$), 3.07-2.08 (m, 4H, -$C\boldsymbol{H}_2$-S-S-$C\boldsymbol{H}_2$-), 4.01-3.84 (m, 2H, -$C\boldsymbol{H}_2$), 4.48-4.31 (m, 2***H***, -CH_2), 5.31 (s, 2H, -$NC\boldsymbol{H}_2$-), 7.69 (d, J = 8.0 Hz, 1H, Ph-H), 7.86 (d, J = 8.0 Hz, 1H, Ph-H), 7.96 (d, J = 8.0 Hz, 1H, Ph-H), 8,24 (d, J = 8.0 Hz, 1H, Ph-H), 8.44 (s, 1H). ^{13}C NMR (100 MHz, $CDCl_3$, ppm): δ 7.63, 31.90, 37.15, 41.12, 50.01, 60.48, 66.42, 67.11, 78.04, 96.36, 98.09, 120.31, 128.22, 128.25, 128.56, 129.31, 130.98, 131.53, 145.63, 146.24, 148.64, 152.23, 153.51, 157.27, 167.42. HRMS (ESI): calcd. for $[C_{25}H_{24}N_2O_7S_2 + H]^+$ 529.1103; found 529.1103. (Figure S9-11).

Synthesis of Cy-S-CPT

CPT-S-OH (120 mg, 0.23 mmol), DMAP (122 mg, 1 mmol) and triphosgene (80 mg, 0.27 mmol) were dissolved in 10 mL anhydrous chloroform, then the mixture was stirred for 30 min in ice bath, the solution changed from white to yellow. CyA-K (260 mg, 0.36 mmol) and DIPEA (0.5 mL) in chloroform (10 mL) were added into mixture. The mixture was stirred for 24 h. Then the solution was poured into 200 mL diether ether, collected the solid and the product was purified by column chromatography (silica gel column, dichloromethane : methanol = 100 : 1) to get a green powder (55 mg), yield 19 %. ^{1}H NMR (400 MHz, $CDCl_3$, ppm): δ 0.98 (t, J = 7.6 Hz, 3H), 1.55-1.46 (m, 6H), 1.94 (s, 12H), 2.06-2.02 (m, 2H), 2.17-2.11 (m, 1H), 2.29-2.20 (m, 1H), 2.77-2.69 (m, 4H), 3.12-3.05 (m, 2H), 3.23-3.15 (m, 2H), 4.36-4.27 (m, 4H), 4.48-4.41 (m, 1H), 4.56-4.48 (m, 1H), 4.73-4.65 (m, 2H), 5.23 (d, J = 14.4 Hz, 1H), 5.31 (s, 1H), 5.37 (d, J = 17.2 Hz, 1H), 5.67 (d, J = 17.2 Hz, 1H), 6.16 (d, J = 14.4 Hz, 2H), 7.51-7.43 (m, 4H), 7.67-7.58 (m, 3H), 7.78 (t, J = 8.0 Hz, 1H), 8.00-7.87 (m, 8H), 8.17-8.08 (m, 3H), 8.47 (s, 1H). ^{13}C NMR (100 MHz, $CDCl_3$, ppm): δ 7.62, 12.71, 14.12, 20.74, 22.63, 24,50, 27.67, 28.04, 31,56, 31,82, 36.65, 37.11, 39.92, 49.23, 50.12, 55.01, 66.46, 67.09, 78.17, 95.17, 99.94, 101.48, 110.63, 110.91, 119.97, 121.79, 122.08, 125.15, 127.54, 127.82, 128.05, 128.09, 128.13, 128.25, 128.45, 129.37, 130.10, 130.74, 130.90, 131.57, 131.92, 133.86, 138.90, 139.10, 140.87, 142.78, 144.35, 145.40, 146.61, 148.71, 152.03, 152.59, 153.50, 157.20. HRMS (ESI): calcd. for $[C_{68}H_{67}O_9N_4S_2]^+$ 1147.4349; found 1147.4360. (Figure S12-14).

Synthesis of CPT-C-OH

The control compound CPT-C was synthesized using the same procedure used in the synthesis of CPT-S-OH. ^{1}H NMR (400 MHz, $CDCl_3$, ppm): δ 1.00 (t, J = 7.6 Hz, 3H), 1.44-1.34 (m, 4H), 1.55-1.51 (m, 2H), 1.73-1.65 (m, 2H), 2.34-2.24 (m, 1H), 2.21-2.12 (m, 1H), 3.60 (t, J = 6.4 Hz, 2H), 4.20-4.07 (m, 2H), 5.31 (s, 2H), 5.40 (d, J = 17.2 Hz, 1H), 5.70 (d, J = 17.2 Hz, 1H), 7.36 (s, 1H), 7.68 (t, J = 8.0 Hz, 1H), 7.95 (d, J = 8.4 Hz, 1H), 8.23 (d, J = 8.4 Hz, 1H), 8.41 (s, 1H). ^{13}C NMR (100 MHz, $CDCl_3$, ppm): δ 7.64, 25.20, 25.34, 28.34, 31.96, 32.41, 49.98, 62.57, 67.07, 69.07, 77.65, 96.15, 120.31, 128.13, 128.19, 128.24, 129.53, 130.80, 131.30, 145.88, 146.34, 148.81, 152.33, 153.81, 167.53. HRMS (ESI): calcd.

for [$C_{27}H_{28}N_2O_7$ + H]$^+$ 493.1975; found 493.1981. (Figure S15-17).

Synthesis of Cy-C-CPT

The control compound CPT-C-CPT was synthesized using the same procedure used in the synthesis of CPT-S-CPT. ^{1}H NMR (400 MHz, $CDCl_3$, ppm): δ 0.96 (t, J = 7.6 Hz, 3H), 1.55-1.48 (m, 10H), 1.81-1.75 (m, 2H), 1.89-1.85 (m, 2H), 1.93 (s, 12H), 2.04-2.00 (m, 2H), 2.11-2.08 (m, 1H), 2.25-2.20 (m, 1H), 2.76-2.71 (m, 4H), 4.15-4.10 (m, 1H), 4.26-4.21 (m, 1H), 4.37-4.31 (m, 6H), 5.31-5.20 (m, 2H), 5.37 (d, J = 17.2 Hz, 1H), 5.67 (d, J = 17.2 Hz, 1H), 6.19 (d, J = 14.2 Hz, 2H), 7.30 (s, 1H), 7.49-7.44 (m, 4H), 7.60 (t, J = 8.0 Hz, 2H), 7.66 (t, J = 8.0 Hz, 1H), 7.81 (t, J = 8.0 Hz, 1H), 7.89 (d, J = 14.2 Hz, 2H), 7.98-7.93 (m, 5H), 8.06 (d, J = 8.4 Hz, 2H), 8.16 (d, J = 8.4 Hz, 1H), 8.42 (s, 1H). ^{13}C NMR (100 MHz, $CDCl_3$, ppm): δ 7.61, 12.71, 14.13, 20.78, 24.46, 25.32, 25.49, 27.55, 27.63, 28.58, 28.65, 29.69, 30.11, 31.88, 39.91, 50.02, 50.90, 67.10, 68.71, 69.60, 77.79, 95.88, 100.05, 110.65, 120.13, 121.90, 122.14, 125.11, 127.80, 128.06, 128.17, 128.41, 129.37, 130.17, 131.45, 131.90, 133.66, 138.87, 139.20, 145.59, 146.45, 148.74, 152.15, 152.75, 153.82, 157.23, 158.53. HRMS (ESI): calcd. for [$C_{70}H_{71}O_9N_4$]$^+$ 1111.5221; found 1111.5216. (Figure S18-20).

Cell lines

Human breast carcinoma cell BCap-37 and Madin-Darby canine kidney cell MDCK were purchased from the American Type Culture Collection (Manassas. VA). BCap-37 cell line was maintained in RPMI 1640 (Gibco) supplemented with 10% foetal bovine serum (FBS, Gibco) and 1% penicillin/streptomycin (Gibco) in a humidified atmosphere of 5% CO_2 at 37 °C. MDCK cells were grown in DMEM (Gibco) containing the same FBS and antibiotics.

Animals

All animal studies were performed according to the guidelines established by the Animal Care and Use Committee of Zhejiang University. Female BALB/c homozygous athymic nude mice at 8-weeks old (average 20 g) were obtained from Silaike Jingda Co., Ltd (China) and maintained under standard conditions.

Encapsulation of Cy-S-CPT and Cy-C-CPT

The prodrug-loaded nanoparticles were prepared using the method reported by Sun[1] with slight modifications. Briefly, Cy-S-CPT or Cy-C-CPT (1 mg) and PEG5000-PLA3000 (9 mg) (weight ratio = 1:9) were dissolved in 2 mL dichloromethane with stirring in a round bottom flask. A thin film was formed by rotary evaporation and completely dried under vacuum. PBS solution (1 mL) was dropwise added into the flask with vigorous stirring, and then stirred for another 2 h to form the micelle solution. After removing the unencapsulated prodrug by a 0.22 μm filtrator, the encapsulated prodrug concentration in the filtrate was determined by HPLC (Waters, with 2998 detector). Drug loading content and encapsulation efficiency were calculated using the following equations:

$$Drug\ loading\ content\ \% = \frac{Weight\ of\ the\ encapsulated\ prodrug}{Weight\ of\ the\ nanoparticles} \times 100\% \qquad (1)$$

$$Encapsulation\ efficiency\ \% = \frac{Weight\ of\ the\ encapsulated\ prodrug}{Weight\ of\ the\ feeding\ prodrug} \times 100\% \qquad (2)$$

A dynamic laser scattering spectrometer (DLS, nano series ZEN3600, Malvern Instruments Ltd., UK) was employed to measure the sizes and zeta potentials of PEG-PLA nanoparticles loaded with the prodrugs, while the nanoparticle concentrations were 10 mg/mL in the samples.

***In vitro* cytotoxicity assay**

3-(4,5-Dimethythiazol-2-yl)-2,5-diphenyltetrazolium bromide (MTT) assays were used to assess the cytotoxicity of the prodrugs on BCap-37 and MDCK cell lines. Briefly, cells were seeded in 96 well plates at a density of 5000 cells per well and incubated overnight. Cells were exposed to serial dilutions of drugs and cultivated for another 48 h, then replaced by fresh medium containing 0.75 mg/mL MTT. After 3 h incubation, the yellow tetrazolium salt (MTT) was metabolized into dark blue formazan crystals, and the MTT medium

solution was carefully removed. Finally, DMSO was added into the wells and the plate was gently shaken to dissolve the precipitates. The absorbance in each well was determined at 562 nm with a microplate spectrophotometer (Molecular Devices, SpectraMax M2e, USA). Cell viability was calculated as the ratio of absorbance of the wells incubated with drug to that of the wells incubated with culture medium.

Cellular uptake and intracellular localization observation

Tumor cells were cultured in glass-bottom petri dishes at a density of 50,000 cell/mL 24 h before treatment. Cells were exposed to the prodrugs at a final concentration of 10 μM for 2 h. Cell nuclei and lysosomes were respectively stained by DAPI and Lyso Tracker Green for 30 min. After washing with PBS for 3 times, cell images were observed using a confocal laser scanning microscope (CLSM, Nikon-A1 system, Japan). Fluorescence of CyA-K released from Cy-S-CPT or Cy-C-CPT was excited using a 488 nm laser, and the emission wavelength was read from 570-620 nm.

Flow cytometry measurement

Tumor cells were plated onto six-well plates at 50,000 cells/mL (2 mL medium per well), cultured for 24 h, and then incubated with 10 μM prodrugs for different time intervals. The cells were harvested using 0.25% (w/v) trypsin/0.02% (w/v) EDTA, collected by centrifuging at 900 rpm for 5 min, washed twice with PBS, and resuspended. Cellular uptake and activation of prodrugs were analyzed on a BD FACSCalibur flow cytometer (BD Biosciences, San Jose, CA). FL3 channel with a 488 nm argon laser was used to scan the CyA-K fluorescence. Each assay collected 10000 gated events and the data was analyzed using the CellQuest Pro software.

***In vivo* and *ex vivo* imaging of mouse with xenografted tumor**

BCap-37 cells were used to develop the human tumor xenografts. After harvested using trypsin, cells were washed 3 times with PBS and injected subcutaneously into the right flanks of the nude mice (1×10^6 cells/mouse). When the tumor volume reached 200 mm^3 by average, the prodrugs loaded in nanoparticles were intravenously injected via tail vein at a

CPT-equivalent dose of 1.75 mg/kg. Whole body optical imaging was scanning with M-MSI-EX small-animal imaging system (CRI) at 0-24 h after injection. The intact prodrugs with emission of 825 nm were excited using a 704 nm laser and the signal was received through a 790 nm filter. The CyA-K with emission of 650 nm was excited using a 523 nm laser and the fluorescence was received through a 630 nm filter. Pentobarbital sodium (80 mg/kg) was used to anesthetize the mice by intraperitoneal injection prior to imaging. For ex vivo imaging, the mice were sacrificed at different times after injection and major organs including heart, kidneys, spleen, lung, stomach, liver and tumors were excised, washed with 0.9% saline and imaged with the same parameter described for *in vivo* imaging.

Plasma pharmacokinetic study

Female ICR mice were randomly divided into 3 groups (n = 3), and intravenously injected with PEG-PLA/Cy-S-CPT, PEG-PLA/Cy-C-CPT and PEG-PLA/CyA-K at a CPT-equivalent dose of 10 μmol/kg respectively. Blood samples were collected into heparinized tubes at different time intervals, centrifuged at 5000 rpm for 10 min at 4 °C, and 100 μL of its supernate plasma was mixed with 900 μL acetonitrile to precipitate all the proteins. After centrifuged, the organic layer was collected. One part of 50 μL acetonitrile solution was acidized using hydrochloric acid and tested by HPLC to determine the CPT concentration; and rest 800 μL acetonitrile solution were concentrated and determine both prodrug level and CyA-K level using HPLC. The concentrations of intact prodrugs in blood plasma were determined by using a Waters 2998 detector with the absorption wavelength of 750 nm; CPT concentration was monitored using the same detector with the absorption wavelength of 360 nm; and CyA-K concentration was determined by a Waters 2475 detector with excitation of 530 nm and emission wavelength read at 650 nm. Standard curves of every components were constructed previously, which were used to determine the exact plasma concentration.

Tumor section and fluorescence imaging analysis

The prodrugs loaded in nanoparticles were intravenously injected into the nude mice with xenografted BCap-37 tumor at a CPT-equivalent dose of 1.75 mg/kg. The mice were

sacrificed at different time intervals and the excised tumors were embedded in Tissue-Tek OCT compound, frozen in liquid nitrogen and sectioned into 10-μm thick slices. The fluorescence images of the tumor sections were observed using confocal laser scanning microscope with the same parameter described in the cellular observation.

***In vivo* antitumor studies**

BCap-37 cells (1×10^6) were injected into the right flank of 21 nude mice, all of which developed tumors in 7 d with sizes of 80 mm^3 by average. The mice were randomly assigned into 3 groups (7 mice/group), and treated by PEG-PLA/Cy-S-CPT (CPT equivalent dose of 10 mg/kg), CPT-11 (with the same CPT equivalent dose) and PBS (control group) by i.v. injection every 3 days for 5 times, respectively. Tumor volume (mm^3) was calculated using the formula: tumor volume = (shortest diameter)^2 × (longest diameter) × 0.5. The tumor growth curves were plotted with the average tumor volumes vs days after the first treatment. All the mice were sacrificed 18 days after the first treatment, and the tumors were resected, weighted and fixed in formalin for paraffin embedding. The inhibition rates of tumor growth (IRT) was calculated as follows: IRT = 100% × (mean tumor weight of the control group – mean tumor weight of the experimental group) / mean tumor weight of the control group.

Histological examination of tumor tissues

The excised tumors were fixed using 4% paraformaldehyde buffer, embedded in paraffin and sectioned into 5-um thick slices. The sections were stained with hematoxylin and esion (H&E, Fisher Scientific, USA) for histological examinations, observed under inverted microscope.

Statistical analysis

Data were presented as means ± standard errors. All the statistical analyses were performed using Student's t-test. Differences were considered statistically significant at a level of $p<0.05$, and very significant when $p<0.01$.

Reference

(1) Wu, X. M.; Sun, X. R.; Guo, Z. Q.; Tang, J. B.; Shen, Y. Q.; James, T. D.; Tian, H.; Zhu, W. H. *J. Amer. Chem. Soc.* **2014**, *136*, 3579.

3. Size distribution of PEG-PLA/Cy-S-CPT and PEG-PLA/Cy-C-CPT

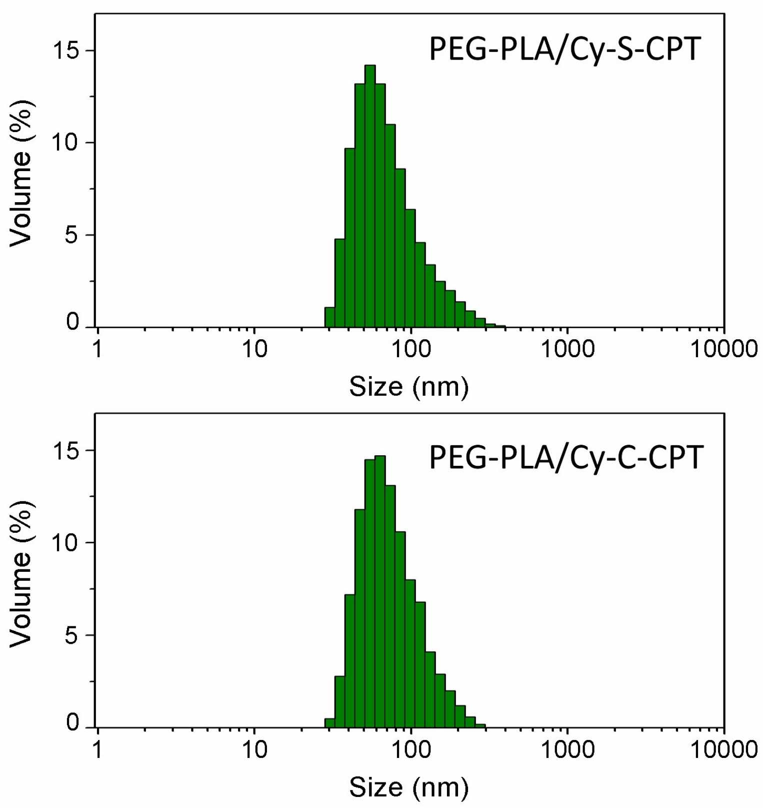

Figure S1. Size distribution of PEG-PLA/Cy-S-CPT and PEG-PLA/Cy-C-CPT nanoparticles measured by DLS.

4. Absorbance ratio of Cy-S-CPT as a function of GSH concentration

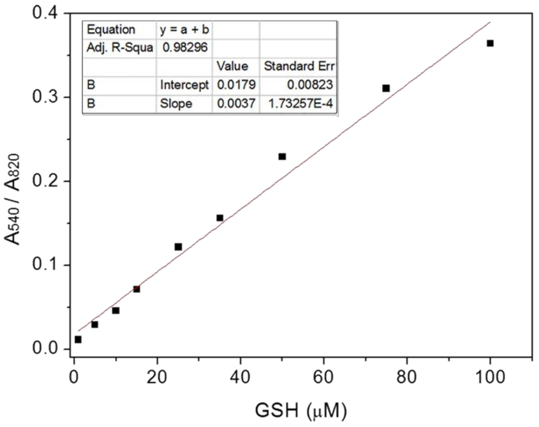

Figure S2. Absorbance ratio ($A_{535\ nm}/A_{820\ nm}$) of Cy-S-CPT (5 μM) as a function of GSH concentration in a mixture solution of DMSO/PBS (40/60, pH = 7.4, 10 mM) at temperature of 37 ^{o}C.

5. Normalized absorption and emission spectra of CyA-K and Cy-S-CPT

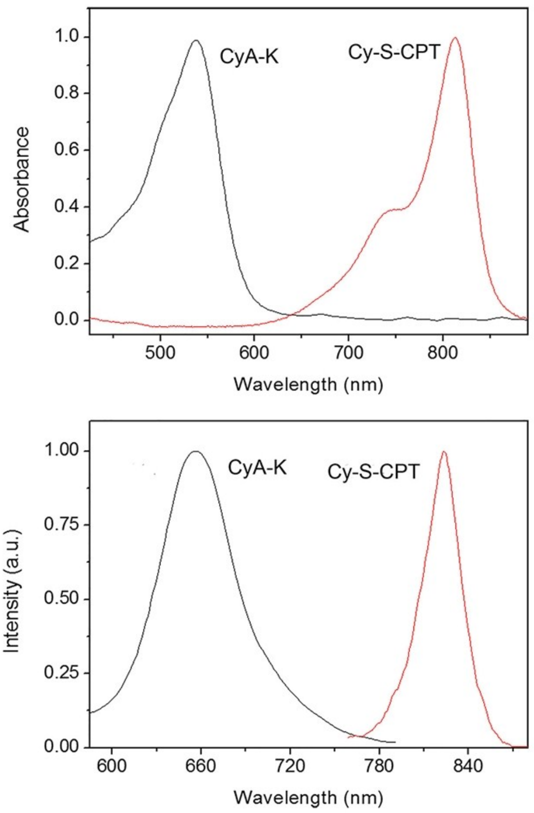

Figure S3. Normalized absorption and emission spectra of CyA-K (5 μM) and Cy-S-CPT (5 μM) in solution as a function of GSH concentration in a mixture solution of DMSO/PBS (40/60, pH = 7.4, 10 mM).

6. Fluorescence spectra of Cy-C-CPT with and without GSH

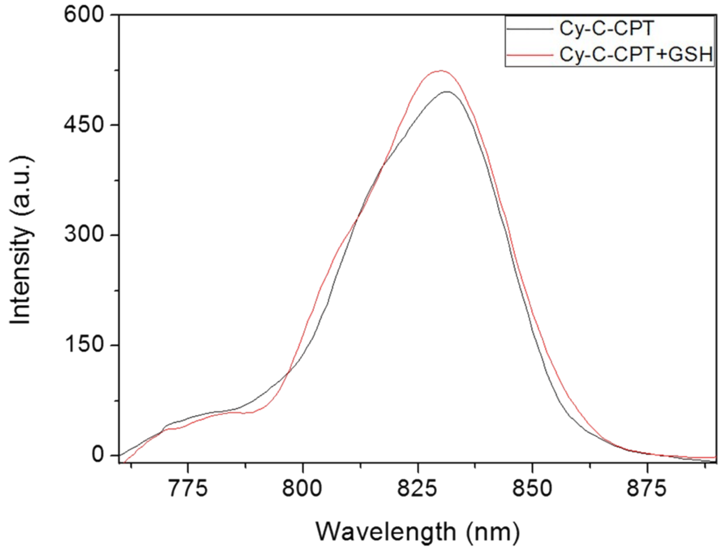

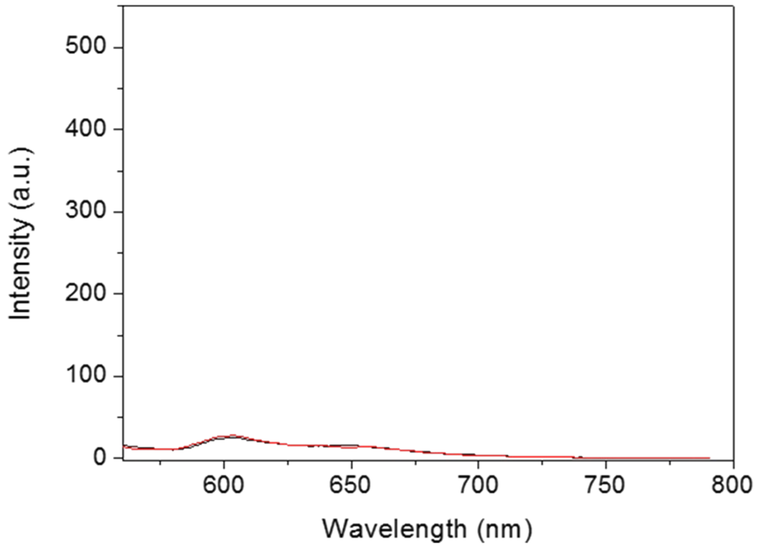

Figure S4. Fluorescence spectra of Cy-C-CPT (5 μM) in a mixed solution of DMSO: PBS (4 : 6, pH = 7.4, 0.01 M) with and without 250 μM of GSH, λ_{ex} = 750 nm (Top) and λ_{ex} = 530 nm (Bottom). Each spectrum was recorded 1 h after exposure at 37 °C.

7. HRMS spectrum of Cy-S-CPT with 50 equiv of GSH

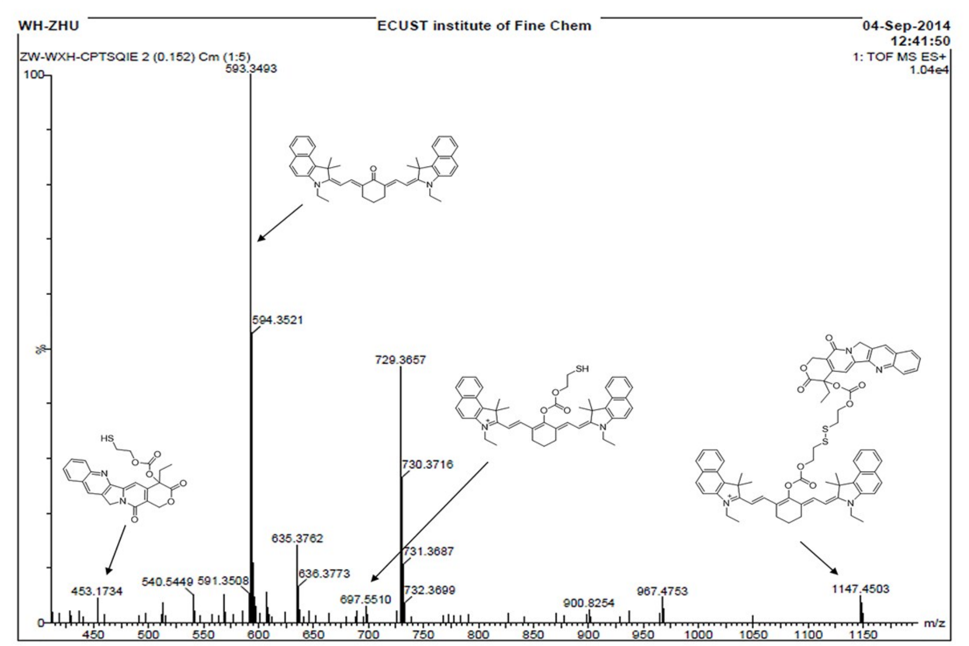

Figure S5. HRMS spectrum of the products from the reaction of Cy-S-CPT (10 μM) in a mixed solution of DMSO: PBS (4 : 6, pH = 7.4, 0.01 M) with 50 equiv of GSH. Spectrum was obtained 1 h after exposure at 37 °C.

8. Fluorescence response of Cy-S-CPT with various thiol-containing structures and amino acids

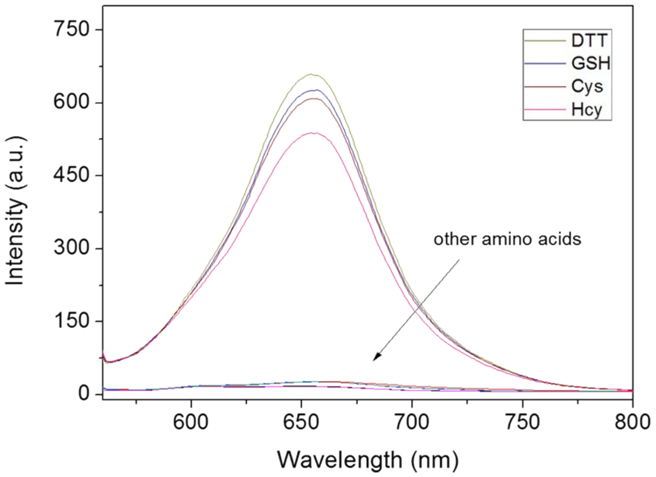

Figure S6. Fluorescence response of Cy-S-CPT (5 μM) upon addition of 250 μM GSH, GSH, Hcy, Cys, DTT and other amino acids inculding Ala, Arg, Asn, Asp, Gln, Glu, Gly, His, Ile, Leu, Phe, Pro, Thr, Tro, Tyr, Val (250 μM) in a mixture solution of DMSO/PBS (40/60, pH = 7.4, 10 mM).

9. Intestinal wall and intestinal contents images of mice injected prodrugs

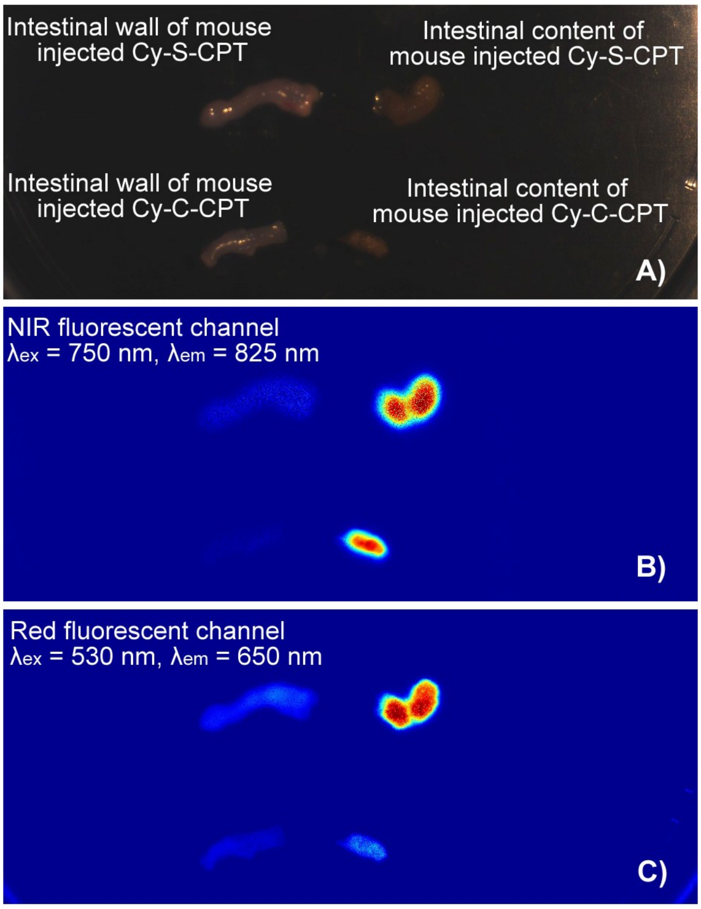

Figure S7. Bright field image (A) and fluorescence images (B for intact prodrug, λ_{ex} = 750 nm, λ_{em} = 825 nm; C for CyA-K, λ_{ex} = 530 nm, λ_{em} = 650 nm) of intestinal wall (left) and intestinal contents (right) for mice injected with PEG-PLA/Cy-S-CPT and PEG-PLA/Cy-C-CPT after anatomy at 4 h after injection.

10. Prodrug penetration kinetics in tumor

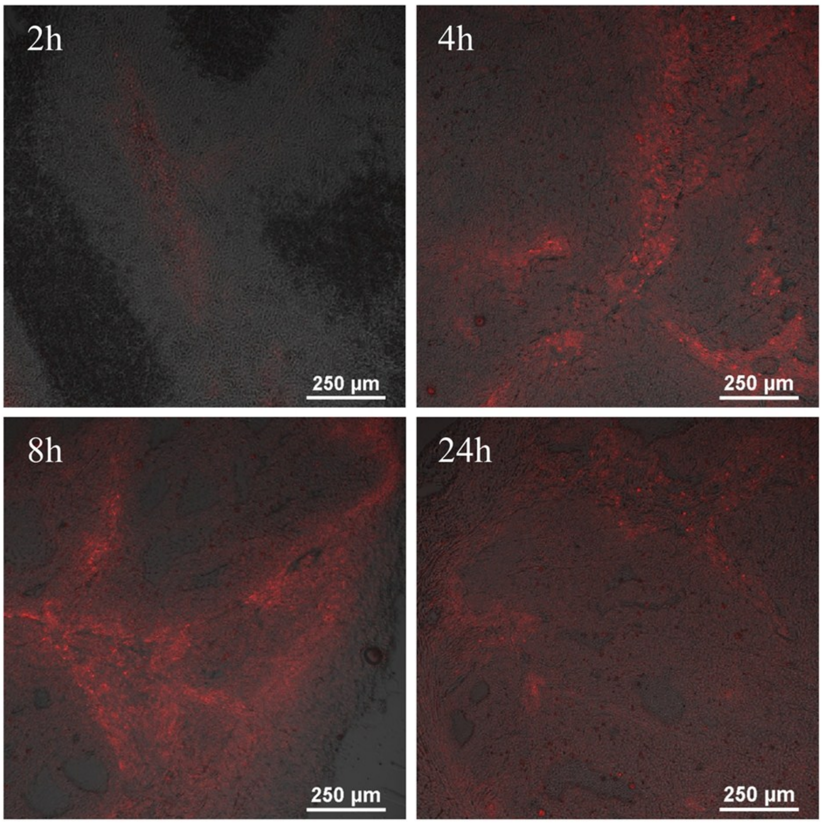

Figure S8. The representative confocal images obtained under scanning of a complete tumor tissue slides sectioned from the tumors treated by PEG-PLA/Cy-S-CPT at different time intervals. Red signal indicate the fluorescence from CyA-K (λ_{ex}=530 nm, λ_{em}=650 nm) that represent the release of the prodrug.

11. ^{1}H and ^{13}C NMR, and HRMS

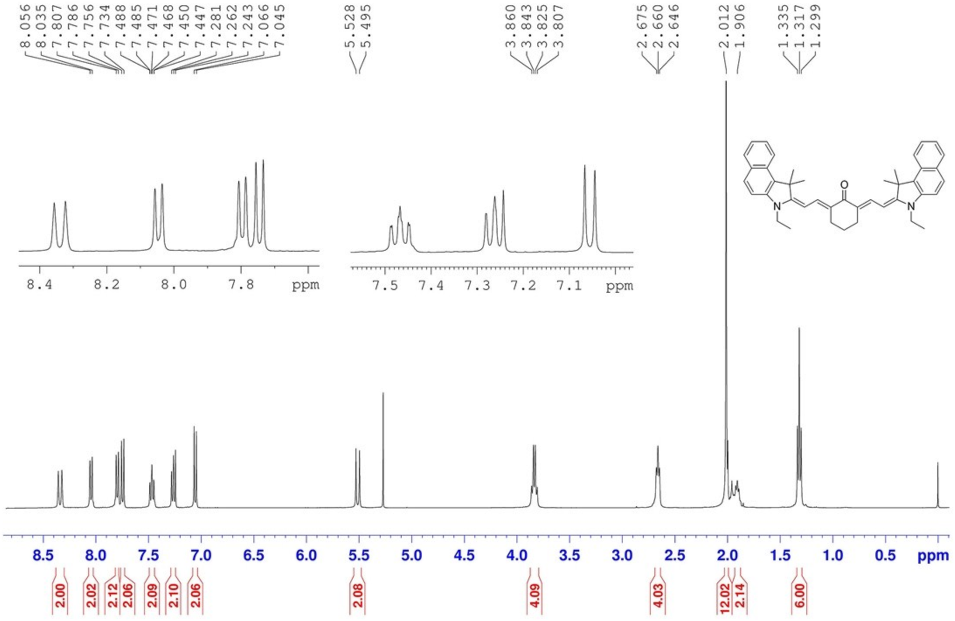

Figure S9. ^{1}H NMR of CyA-K

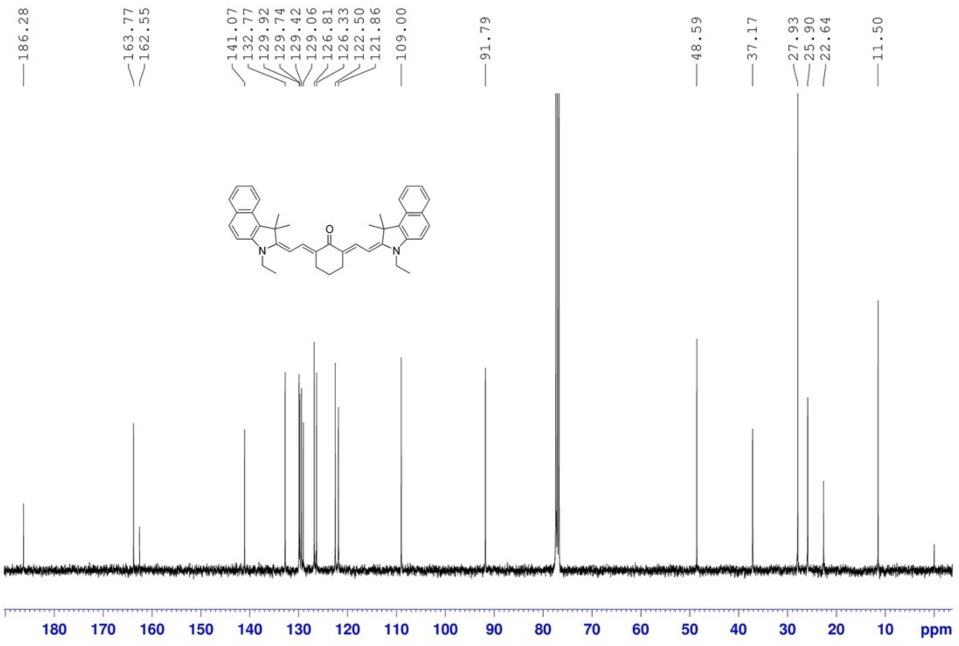

Figure S10. ^{13}C NMR of CyA-K

Elemental Composition Report **Page 1**

Single Mass Analysis
Tolerance = 50.0 mDa / DBE: min = -1.5, max = 100.0
Element prediction: Off
Number of isotope peaks used for i-FIT = 2

Monoisotopic Mass, Even Electron Ions
29 formula(e) evaluated with 4 results within limits (up to 1 closest results for each mass)
Elements Used:
C: 0-46 H: 0-120 N: 2-2 O: 0-3

WH-ZHU ECUST institute of Fine Chem 09-Oct-2012 09:59:37

ZWH-WXH-01 7 (0.299) Cm (7:11) 1: TOF MS ES+ 7.00e+002

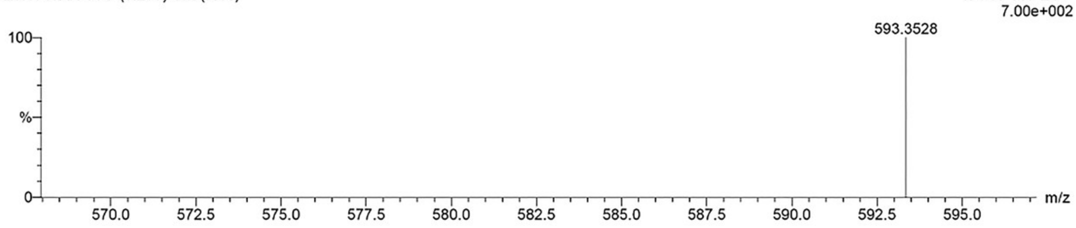

Minimum:				-1.5			
Maximum:		50.0	50.0	100.0			
Mass	Calc. Mass	mDa	PPM	DBE	i-FIT	i-FIT (Norm)	Formula
593.3528	593.3532	-0.4	-0.7	21.5	9.8	0.0	C42 H45 N2 O

Figure S11. TOF MS ES of CyA-K

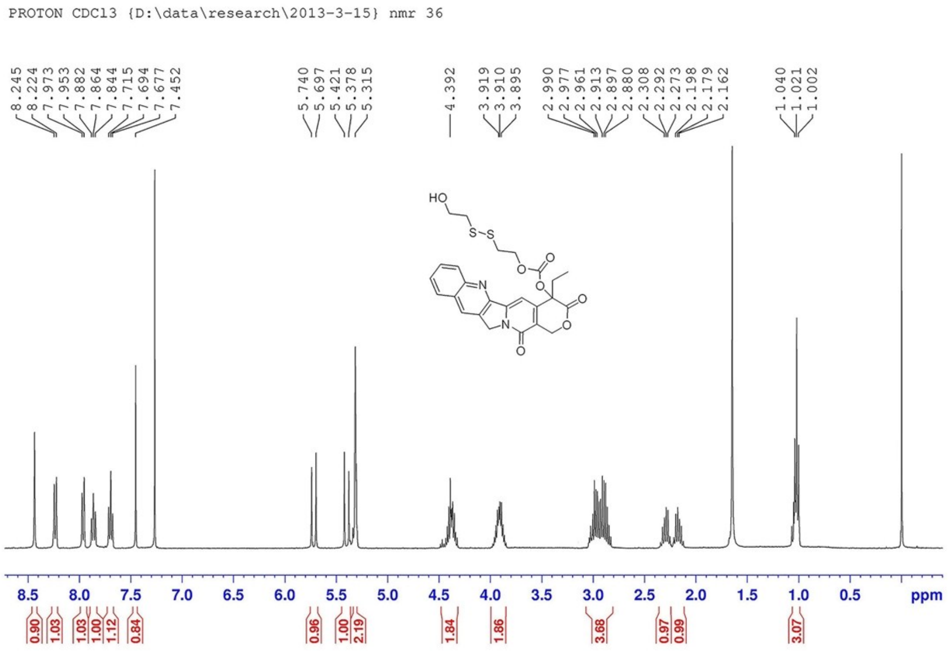

Figure S12. ^{1}H NMR of CPT-S-OH

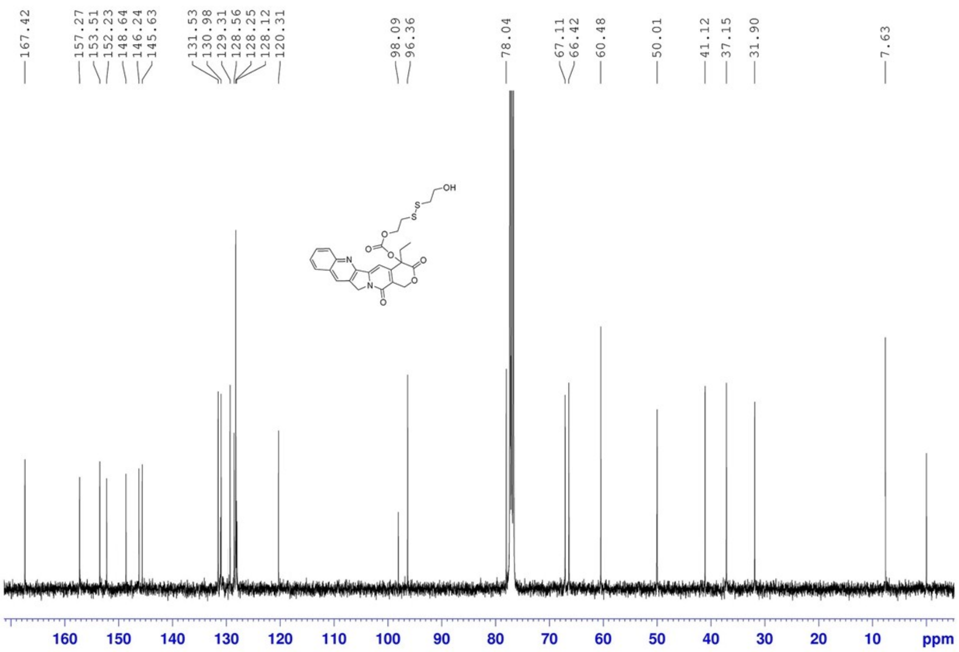

Figure S13. ^{13}C NMR of CPT-S-OH

Elemental Composition Report **Page 1**

Single Mass Analysis
Tolerance = 30.0 mDa / DBE: min = -1.5, max = 100.0
Element prediction: Off
Number of isotope peaks used for i-FIT = 2

Monoisotopic Mass, Even Electron Ions
153 formula(e) evaluated with 6 results within limits (up to 1 best isotopic matches for each mass)
Elements Used:
C: 0-25 H: 0-30 N: 0-6 O: 0-7 S: 0-2

WH-ZHU ECUST institute of Fine Chem 04-Sep-2014 12:29:51

ZW-WXH-CPTS 60 (1.921) Cm (59:61) 1: TOF MS ES+ 7.83e+002

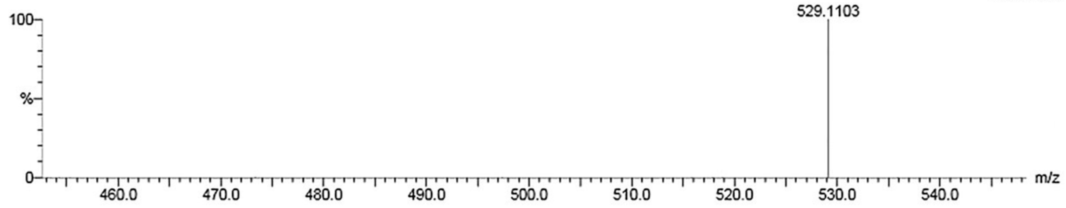

Minimum:				-1.5			
Maximum:		30.0	50.0	100.0			
Mass	Calc. Mass	mDa	PPM	DBE	i-FIT	i-FIT (Norm)	Formula
529.1103	529.1103	0.0	0.0	14.5	7.1	0.0	C25 H25 N2 O7 S2

Figure S14. TOF MS ES of CPT-S-OH

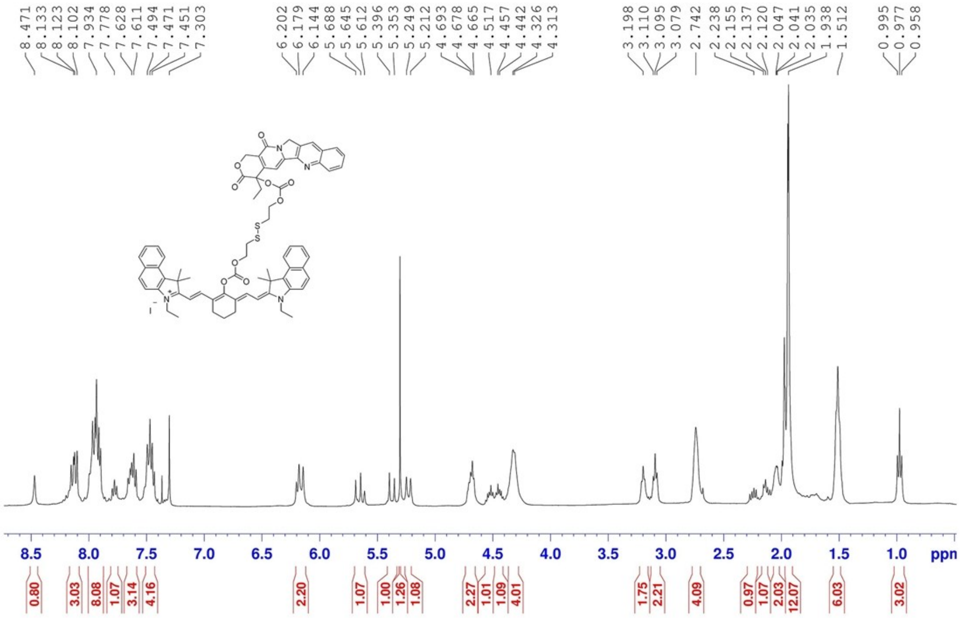

Figure S15. ^{1}H NMR of Cy-S-CPT

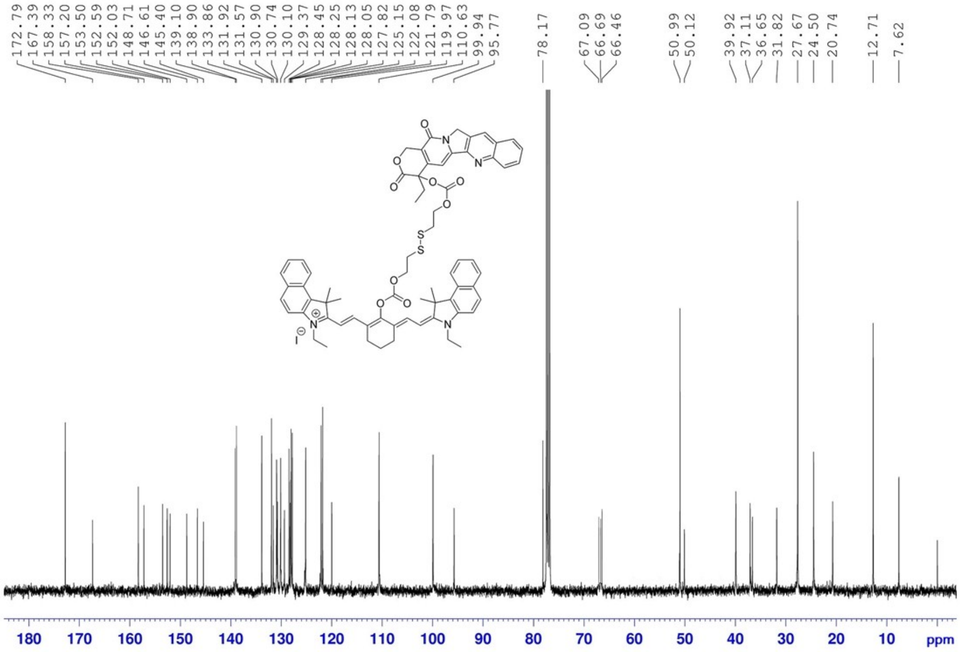

Figure S16. ^{13}C NMR of Cy-S-CPT

Elemental Composition Report Page 1

Single Mass Analysis
Tolerance = 30.0 mDa / DBE: min = -1.5, max = 100.0
Element prediction: Off
Number of isotope peaks used for i-FIT = 2

Monoisotopic Mass, Even Electron Ions
112 formula(e) evaluated with 6 results within limits (up to 1 best isotopic matches for each mass)
Elements Used:
C: 0-71 H: 0-71 N: 0-4 O: 0-10 S: 0-2

WH-ZHU ECUST institute of Fine Chem 23-Jul-2014 15:57:17
ZWH-WXH-723-3 23 (0.791) Cm (22:24) 1: TOF MS ES+ 1.88e+003

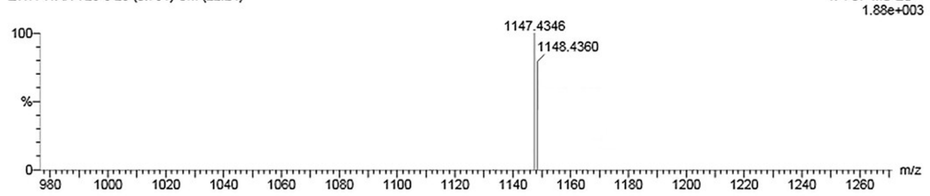

Minimum:				-1.5			
Maximum:		30.0	50.0	100.0			
Mass	Calc. Mass	mDa	PPM	DBE	i-FIT	i-FIT (Norm)	Formula
1147.4346	1147.4349	-0.3	-0.3	37.5	9.0	0.0	C68 H67 N4 O9 S2

Figure S17. TOF MS ES of Cy-S-CPT

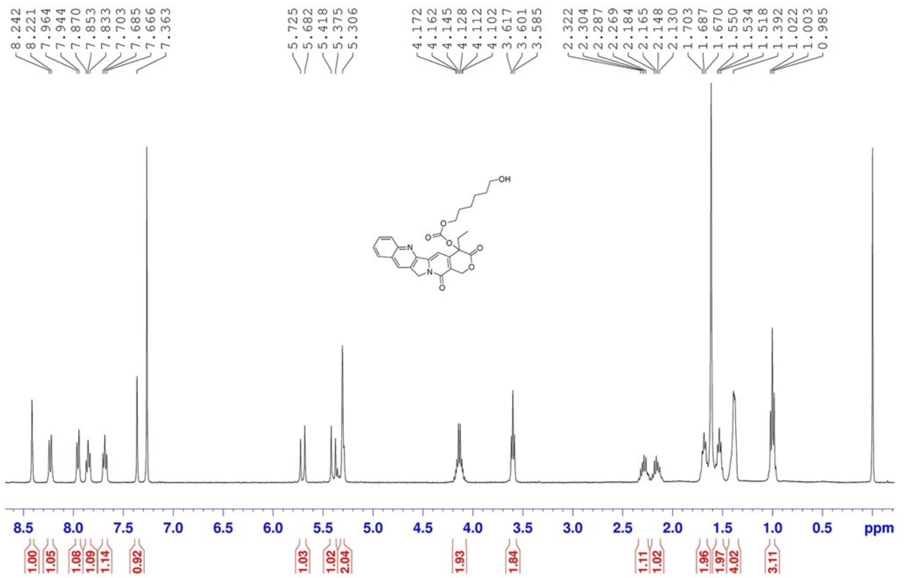

Figure S18. ^{1}H NMR of Cy-C-OH

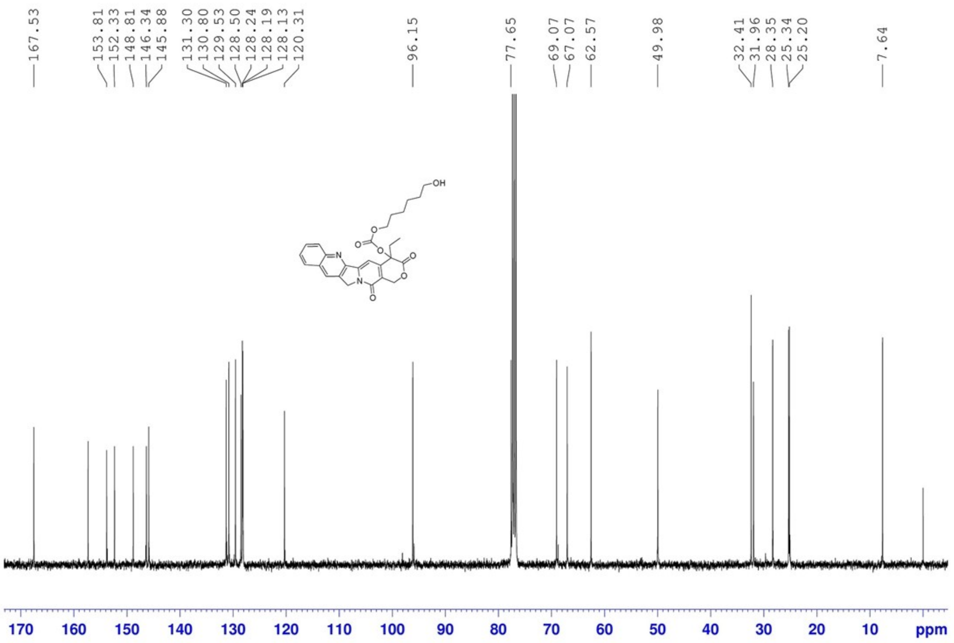

Figure S19. ^{13}C NMR of Cy-C-OH

Elemental Composition Report **Page 1**

Single Mass Analysis
Tolerance = 30.0 mDa / DBE: min = -1.5, max = 100.0
Element prediction: Off
Number of isotope peaks used for i-FIT = 2

Monoisotopic Mass, Even Electron Ions
52 formula(e) evaluated with 5 results within limits (up to 1 best isotopic matches for each mass)
Elements Used:
C: 0-27 H: 0-30 N: 0-6 O: 0-7

WH-ZHU ECUST institute of Fine Chem 04-Sep-2014 12:35:48

ZW-WXH-CPTC 31 (1.036) Cm (29:32) 1: TOF MS ES+ 1.89e+003

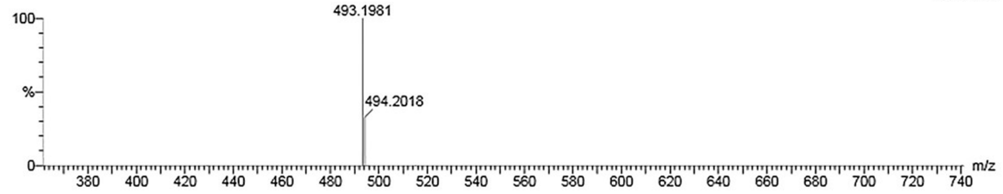

Minimum:				-1.5			
Maximum:		30.0	50.0	100.0			
Mass	Calc. Mass	mDa	PPM	DBE	i-FIT	i-FIT (Norm)	Formula
493.1981	493.1975	0.6	1.2	14.5	7.0	0.0	C27 H29 N2 O7

Figure S20. TOF MS ES of Cy-C-OH

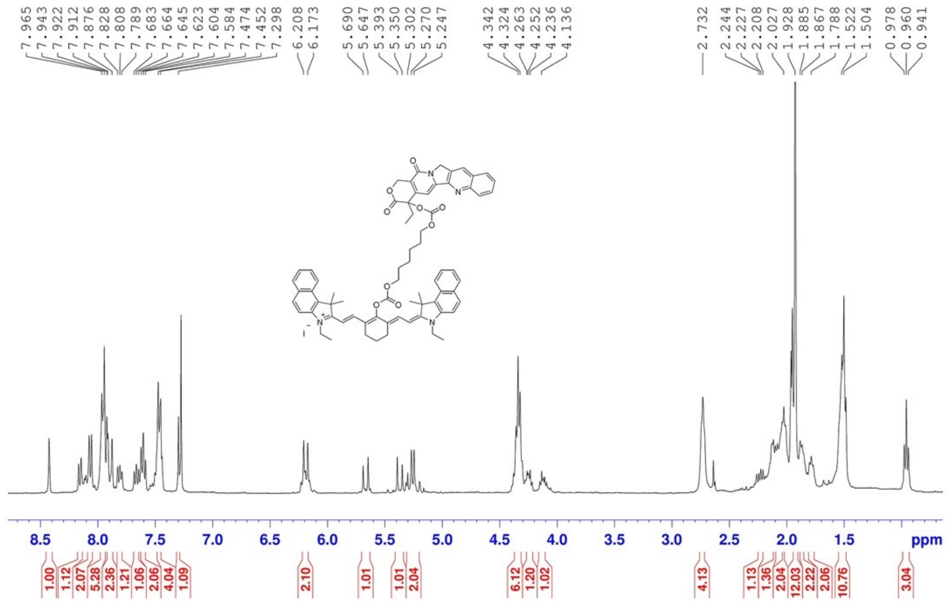

Figure S21. ^{1}H NMR of Cy-C-CPT

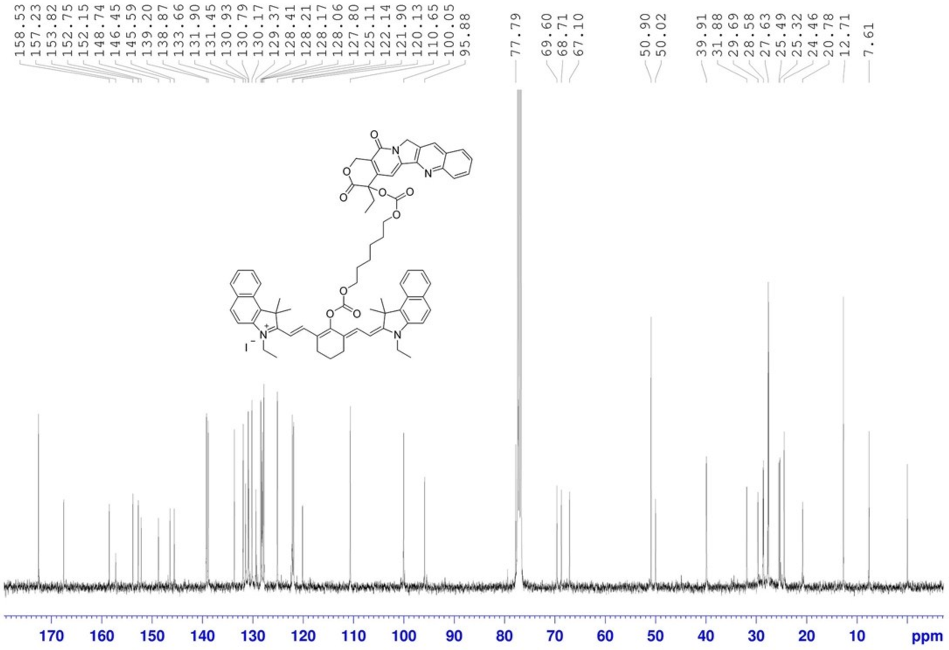

Figure S22. ^{13}C NMR of Cy-C-CPT

Elemental Composition Report **Page 1**

Single Mass Analysis
Tolerance = 30.0 mDa / DBE: min = -1.5, max = 100.0
Element prediction: Off
Number of isotope peaks used for i-FIT = 2

Monoisotopic Mass, Even Electron Ions
31 formula(e) evaluated with 1 results within limits (up to 1 best isotopic matches for each mass)
Elements Used:
C: 0-70 H: 0-71 N: 0-4 O: 0-9

WH-ZHU ECUST institute of Fine Chem 23-Jul-2014 15:37:27

ZWH-WXH-723-1 19 (0.668) Cm (18:20) 1: TOF MS ES+ 1.79e+003

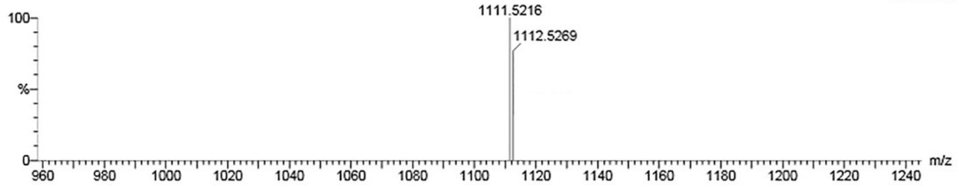

Minimum:				-1.5			
Maximum:		30.0	50.0	100.0			
Mass	Calc. Mass	mDa	PPM	DBE	i-FIT	i-FIT (Norm)	Formula
1111.5216	1111.5221	-0.5	-0.4	37.5	9.5	0.0	C70 H71 N4 O9

Figure S23. TOF MS ES of Cy-C-CPT
